# Cationic Geminoid Peptide Amphiphiles Inhibit DENV2 Protease, Furin, and Viral Replication

**DOI:** 10.3390/molecules27103217

**Published:** 2022-05-17

**Authors:** Mark Damen, Mario A. Izidoro, Debora N. Okamoto, Lilian C. G. Oliveira, Helene I. V. Amatdjais-Groenen, Stijn F. M. van Dongen, Koen W. R. van Cleef, Ronald P. van Rij, Cindy E. J. Dieteren, Daniel Gironés, Bernd N. M. van Buuren, Byron E. E. Martina, Albert D. M. E. Osterhaus, Luiz Juliano, Bob J. Scholte, Martin C. Feiters

**Affiliations:** 1 Department of Organic Chemistry, Institute for Molecules and Materials, Faculty of Science, Radboud University, Heyendaalseweg 135, 6525 AJ Nijmegen, The Netherlands; mdamenster@gmail.com (M.D.); h.amatdjais-groenen@science.ru.nl (H.I.V.A.-G.); science@stijnvandongen.nl (S.F.M.v.D.); 2 Department of Biophysics, Escola Paulista de Medicina, Universidade Federal de São Paulo (UNIFESP), Rua Três de Maio, 100, São Paulo 04044-020, Brazil; mizidoro@gmail.com (M.A.I.); deboraok@yahoo.com.br (D.N.O.); lilian_cgo@yahoo.com.br (L.C.G.O.); 3 Laboratory of Spectrometry, Beneficent Association of Blood Collection, Associação Beneficente de Coleta de Sangue (COLSAN), Av. Jandira 1260, São Paulo 04080-006, Brazil; 4 Department of Medical Microbiology, Radboud Institute for Molecular Life Sciences, Radboud University Medical Centre, 6500 HB Nijmegen, The Netherlands; koenvancleef@hotmail.com (K.W.R.v.C.); ronald.vanrij@radboudumc.nl (R.P.v.R.); 5 Protinhi Therapeutics, Transistorweg 5, 6534 AT Nijmegen, The Netherlands; c.dieteren@protinhi.com (C.E.J.D.); d.girones@protinhi.com (D.G.); b.vanbuuren@protinhi.com (B.N.M.v.B.); 6 Department of Viroscience, Erasmus Medical Centre, 3015 GD Rotterdam, The Netherlands; b.martina@artemisonehealth.com; 7 Artemis Bio-Support, Molengraaffsingel 10, 2629 JD Delft, The Netherlands; albert.osterhaus@tiho-hannover.de; 8 Research Center for Emerging Infections and Zoonoses, University of Veterinary Medicine Hannover, 30559 Hannover, Germany; 9 Department of Cell Biology, Erasmus Medical Centre, 3000 CA Rotterdam, The Netherlands; 10 Pediatric Pulmonology, Erasmus Medical Centre, 3000 CA Rotterdam, The Netherlands

**Keywords:** amphiphiles, drug discovery, inhibitors, membrane proteins, peptides

## Abstract

Dengue is an important arboviral infectious disease for which there is currently no specific cure. We report gemini-like (geminoid) alkylated amphiphilic peptides containing lysines in combination with glycines or alanines (C_15_H_31_C(O)-Lys-(Gly or Ala)_n_Lys-NHC_16_H_33_, shorthand notation C_16_-KX_n_K-C_16_ with X = A or G, and *n* = 0–2). The representatives with 1 or 2 Ala inhibit dengue protease and human furin, two serine proteases involved in dengue virus infection that have peptides with cationic amino acids as their preferred substrates, with IC_50_ values in the lower µM range. The geminoid C_16_-KAK-C_16_ combined inhibition of DENV2 protease (IC_50_ 2.3 µM) with efficacy against replication of wildtype DENV2 in LLC-MK2 cells (EC_50_ 4.1 µM) and an absence of toxicity. We conclude that the lysine-based geminoids have activity against dengue virus infection, which is based on their inhibition of the proteases involved in viral replication and are therefore promising leads to further developing antiviral therapeutics, not limited to dengue.

## 1. Introduction

Dengue is responsible for close to 400 million infections worldwide per year, of which 25,000 are fatal [1,2]. Currently, there is no specific therapy available for dengue, and vaccine development has been proven difficult, exemplified by its yielding only limited immunity while its safety is under debate (see e.g., [3,4,5]). Upon infection by dengue and many other arboviruses, including pathogenic members of the *Flaviviridae* family (West Nile, Zika, Yellow Fever), the viral RNA is translated into a polyprotein, which is cleaved [6,7] into structural (C, prM, E) and non-structural (NS) proteins by the concomitant action of viral and host proteases. The active site of the dengue virus protease is in the N-terminal part of NS3, which is a serine protease with a catalytic triad of Asp75-His51-Ser135 for dengue virus serotype 2 (DENV2) but requires a conserved domain of NS2B to form a fully active heterodimer with complete substrate recognition [8,9]. The functional similarity between the NS2B/NS3 proteases from the four genetically and antigenically distinct serotypes was shown previously [10], and the DENV2 protease can therefore be considered a good model for all DENV proteases. In addition to its role in virus polyprotein processing and viral replication, DENV protease cleaves the STING (stimulator of interferon genes) protein, which resides in the endoplasmic reticulum and is involved in innate immune signalling. STING cleavage results in the inhibition of the type-I IFN (interferon) response allows the virus to evade the innate immune system; as a consequence, inactivation of STING by viral protease results in increased DENV replication [11,12]. Moreover, DENV protease may also exacerbate DENV pathology because it cleaves IκB (inhibitory proteins) in endothelial cells, thereby activating the transcription factor NF-κB, which results in endothelial cell death and has been suggested to cause the transition from dengue fever to the potentially lethal dengue hemorrhagic fever [13].

A host protease involved in DENV replication, more specifically the maturation of prM to give infectious virus particles [14], is human furin, a proprotein convertase (PC), also known as PACE (Paired Basic Amino Acid Cleaving Enzyme), which, similar to the DENV protease, is a serine protease. Although the inhibition of furin might lead to adverse side effects since it has important physiological functions in endogenous protein maturation [15], it is also considered a relevant target for antiviral therapy for the dengue virus [16]. The viral proteases and furin are important targets for antiviral drug development [17,18,19], and protease inhibitors are already used in the clinic against hepatitis C virus and human immunodeficiency virus (HIV) [20,21]. Inhibition of the DENV protease is therefore considered to be of high interest for antiviral treatment as well.

The substrate specificities of proteases can be studied with Fluorescence Resonance Energy Transfer (FRET) substrates, where the fluorescence of the N-terminal Abz (aminobenzoyl) group is quenched by a C-terminal 3-nitrotyrosine [22] or EDDnp (ethylenediamine-dinitrofluorophenyl) group [23] until the peptide is cleaved, or a peptide with C-terminal 7-amino-4-methyl coumarin amide (MCA) [23,24,25] that releases a fluorescent group upon cleavage. Such studies have shown that Abz-AKRR↓SQ-EDDnp is a good substrate for DENV2 protease [23], while furin prefers the acetyl (Ac)/MCA derivative Ac-RVRR-MCA [24]. These observations suggest that DENV2 protease and furin have subtly different preferred peptide sequences as substrates, namely, with cationic residues in positions P_1_-P_2_-P_3_ and P_1_-P_2_-P_4_, respectively, on the N-terminal side of the site of cleavage (↓). Indeed, the dengue viral polyprotein contains a number of these peptide sequences (see [9] for an overview). The peptide 2-Abz-Nle-Lys-Arg-Arg-Ser-Tyr(3-NO_2_)-NH_2_ (hereafter called Tyr(3-NO_2_) substrate), which contains the recognition residues P_4_–P_1_ [22], is a suitable substrate for inhibition studies of DENV2 protease and was applied in the present work for an in-depth investigation of selected inhibitors, while for explorative studies the more readily available MCA derivative of the dipeptide -RR- (Z-RR-MCA, with Z- = PhCH_2_O(CO)-, also known as Cbz-) [23,25] was used; the kinetic parameters for the hydrolysis of this substrate (k_cat_ = 0.11 s^−1^; K_M_ = 247 μM)) were reported in [23].

**Figure 1:**
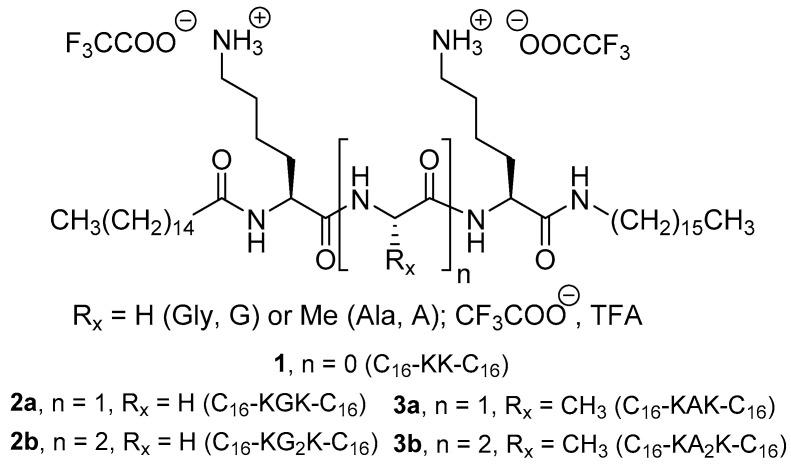
Geminoid structures.

We have developed a novel type of amphiphilic peptide (Figure 1) [26,27] that we have called ‘gemini-like’ or ‘geminoid’ because they can be considered gemini surfactants on the basis of the presence of two alkyl tails and the peptide spacer, but are different from classical geminis [28] due to the asymmetry of the peptide, which has an acyl (fatty acid) and an alkyl (amine) moiety appended to the N- and C-termini, respectively. Cationic representatives of this novel class of compounds, such as oleoyl-Ser-Pro-Lys-Arg-oleyl (ol-SPKR-ol) and analogues with saturated alkyl chains such as palmitoyl-Lys-(Ala or Gly)_n_-Lys-hexadecyl, denoted as n-C_15_H_31_C(O)-K(X)_n_K-(NH)-n-C_16_H_33_ with X = A or G (compounds **1–3**, shorthand representation C_16_-KX_n_K-C_16_), were designed for complexation of polynucleotides and their transfer across biological membranes [27], with the ultimate goal of transfection, gene therapy [29], and RNA inhibition (RNAi) [30]. For such applications, lipids must be cationic to interact with and compensate for the negative charge of the phosphates in the nucleotides, and Lys is preferred as the cationic amino acid over Arg because the positive charge of the latter is permanent, whereas that of the former is pH-dependent, i.e., it is involved in protonation equilibria (procationic), a factor which promotes endosomal escape of the polynucleotide upon uptake in the cell by endocytosis [31].

Because of the preference of DENV protease and furin for substrates with cationic amino acids, we investigated whether geminoids with Lys (compounds **1–3**) could inhibit the activity of these proteases and whether any selectivity could be detected in spite of their similar substrate preferences. Here, we show that geminoids of the C_16_-KA_n_K-C_16_ (**2**) and C_16_-KG_n_K-C_16_ (**3**) series, in particular with A and *n* = 1 (**2a**) or 2 (**2b**), are effective inhibitors of DENV2 protease and the host protease furin and explore their selectivity with another clinically relevant protease, trypsin. The inhibitors are also shown to be active against DENV2 infection in a cellular context at non-toxic concentrations.

## 2. Results

### 2.1. Inhibition of DENV2 Protease and Furin by Geminoids Studied with MCA Substrates

The IC_50_ values of the geminoid peptide amphiphiles **1–3** (Figure 1, with y = 16, R_1_ = n-C_15_H_31_, R_2_ = n-C_16_H_33_) for DENV2 protease and furin with MCA substrates are given in the left part of Table 1 (see Figures S1 and S2 in the Supporting Information for graphical representations). The geminoids with Ala (**2**) were better inhibitors than those with Gly (**3**), and C_16_-KAK-C_16_ was found to be the better inhibitor for DENV2 protease compared to C_16_-KA_2_K-C_16_ (IC_50_ values of 0.66 resp. 0.80 µM), while for furin it was the other way around (IC_50_ values of 3.57 resp. 2.14 µM).

The IC_50_ values determined for inhibition of trypsin for a number of selected geminoids were more than an order of magnitude higher than those for DENV2 protease (Table 1, Figure S3). The serine proteases that are highly susceptible to inhibition by cationic geminoids have a preference for substrates that contain cationic amino acids [23,24] and are active on proteins that are located in the membrane of the endoplasmic reticulum [6].

### 2.2. Effect of Lipid Aggregation on the Inhibition

The inhibition of furin by the C_16_-KG_n_K-C_16_ (**3**) compounds with Ac-RVRR-MCA as the substrate in competitive inhibition experiments had a non-linear dependence on inhibitor concentration (see Figure S4 for the example of **3b**). We observed the following three phases: (i) a decrease in activity by 30–40% in the inhibitor concentration range of 0–4 µM; (ii) a plateau in the region of 4–12 µM; (iii) a steep decrease to full inhibition above 12 µM. We determined the critical micelle or aggregate concentration (CMC) of a number of effective inhibitors by studying the fluorescence of pyrene as a probe (see Figure S5) [32,33]. Because a possible explanation for the multi-phase behaviour would be that the CMC of the geminoid corresponds to the transition between phases (ii) and (iii) and that the last phase represents a very efficient inhibition by inhibitor aggregates, which would imply that the 1st and 2nd phases represent the maximum degree of inhibition attainable with non-aggregated monomer. The CMC values found were, however, all in the order of 10–100 µM (Table 1), which is typical for geminis [28]. They decreased with the length of the spacer, in line with what is observed for gemini surfactants with alkyl spacers with more than 4–6 methylene groups [34], but did not appear to be correlated to the type of amino acid (Ala or Gly) in the spacer. The CMC values of the most effective inhibitors are well above the IC_50_ values for both DENV2 protease and furin for these compounds (Table 1). Although the CMC values are determined in pure water and could be affected by solutes in the various assay buffers, we conclude that micelle formation or aggregation probably does not play a major role in the inhibition assays.

### 2.3. Inhibition of DENV2 Protease by Geminoids Studied with Tyr(3-NO_2_) Substrate

**Figure 2:**
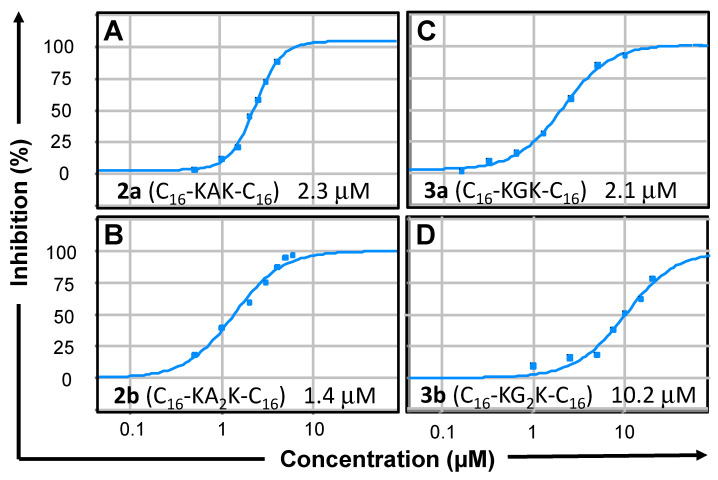
Inhibition of DENV2 protease by geminoids **2** (left: panel (**A**), compound **2a**, panel (**B**), compound **2b**) and **3** (right: panel (**C**), compound **3a**; panel (**D**), compound **3b**). Dose-response curves (average of experiments performed in triplicate) for the biochemical assay with 50 μM Tyr(3-NO_2_) substrate in 50 mM Tris–HCl, pH 9.0, ethylene glycol (10% *v*/*v*), Brij^®^58 (0.0016%). See insets and Table 1 for averaged IC_50_ values from curve fits.

To further explore the dependence of DENV2 protease inhibition on the choice of substrate, a selected group of geminoids was investigated with the aforementioned Tyr(3-NO_2_) substrate [22,35,36,37]. The IC_50_ values determined in this assay (Table 1, Figure 2) showed that the geminoids were effective inhibitors in this assay as well, but contrary to the results with Z-RR-MCA (Table 1), the superiority of the geminoids with Ala residues in the spacer (**2**) over those with Gly (**3**), in particular, **3a** (C_16_-KGK-C_16_), was less pronounced with this substrate; moreover, the order appeared to be reversed, as **2b** (C_16_-KA_2_K-C_16_) was a better inhibitor than **2a** (C_16_-KAK-C_16_).

### 2.4. Effect of Geminoids on DENV2 Replicon Activity in HeLa Cells

**Figure 3:**
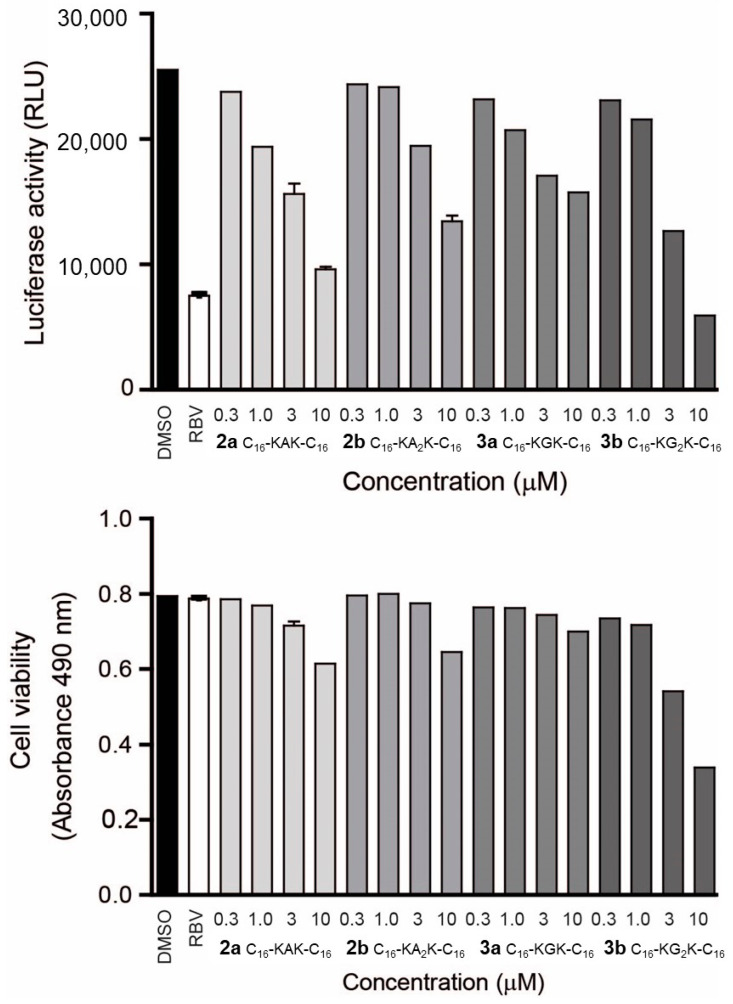
Inhibition of DENV2 replication by geminoids **2a-b** and **3a-b**. DENV2 HeLa replicon cells were treated for 2 days and luciferase activity (**top** panel) and cell viability (MTS assay, absorbance at 490 nm; **bottom** panel) were assessed. Bars represent means and standard deviation of three biological replicates. DMSO, control with only solvent; RBV, ribavirin (10 µM).

To investigate the effect of geminoids on viral replication, we used HeLa cells containing a DENV2 replicon. Instead of the structural proteins of DENV2, the replicon encodes a luciferase reporter that can be used as a readout for DENV2 protease dependent virus replication [38]. Cell viability was assessed on the same cells using a colourimetric assay based on 3-(4,5-dimethylthiazol-2-yl)-5-(3-carboxymethoxyphenyl)-2H-tetrazolium (MTS) reduction, and by light microscopy. The most effective compounds from Table 1 were tested at concentrations ranging from 0.3 to 10 µM, well below their CMC values. **2a** (C_16_-KAK-C_16_) proved most effective in this system, with a 39% reduction of luciferase activity at 3 µM and 62% inhibition at 10 µM (Figure 3, top panel). We cannot exclude that this reduction at the highest concentration tested is partly due to cytotoxicity (Figure 3, bottom panel). Other compounds, **2b** (C_16_-KA_2_K-C_16_) and **3a** (C_16_-KGK-C_16_) (MTS assay >80%, relative to DMSO control) with a similar toxicity profile, were less effective (47% and 38% inhibition at 10 µM, respectively). **3b** (C_16_-KG_2_K-C_16_) induced considerable cytotoxicity, and we thus could not establish DENV2 inhibition by this compound in this assay. With the exception of **3b** (C_16_-KG_2_K-C_16_), these geminoids have a concentration window for which luciferase activity is reduced with cell viability values of >80%. Inhibition of DENV replicon follows the trends in IC_50_ found in the studies on the inhibition of the DENV2 protease construct with the MCA substrate (Table 1). The viability of unmodified HeLa cells after treatment with these compounds was also tested in a separate experiment using the Celltiter Blue Viability Assay (Promega, see Supporting Information) in the concentration range of 0.8–50 μM. No CC_50_ could be calculated at this concentration range (Figure S6), and only **3b** (C_16_-KG_2_K-C_16_) showed slight toxicities at the highest concentration. The geminoids appear to be less toxic to unmodified HeLa cells than to the replicon-containing cells.

### 2.5. Inhibition of DENV2 Replication in LLC-MK2

**Figure 4:**
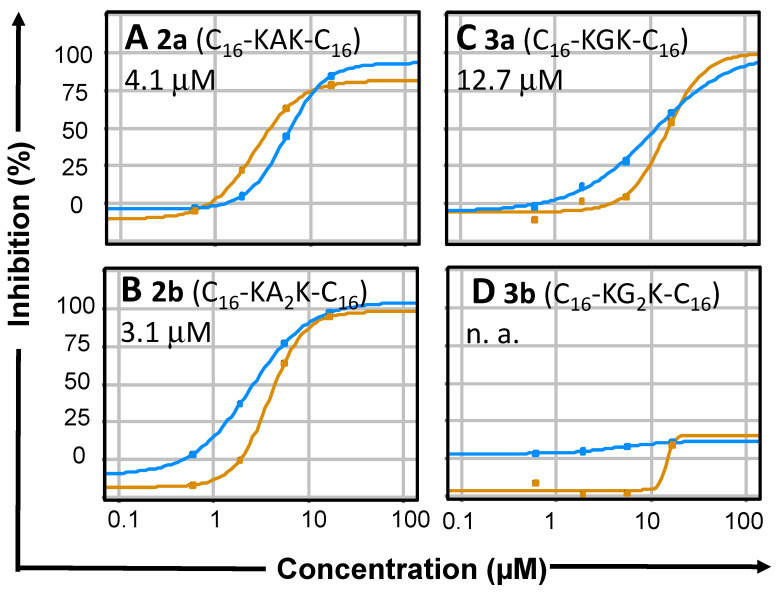
Dose-response curves for the virus infection assay with LLC-MK2 cells for geminoids **2** (left: Panel (**A**), compound **2a**, Panel (**B**), compound **2b**) and **3** (right: Panel (**C**), compound **3a**; Panel (**D**), compound **3b**). The assay, which reports the percentage of infected cells by an immunochemical approach, was performed in duplicate (series-1 and series-2) at 8 concentrations (three-fold dilutions), see Table 1 for averaged EC_50_ values with standard deviation from curve fits; n. a., not active.

To assess the antiviral activity, we studied the inhibitors with wildtype DENV2 (DENV2 NGC) in LLC-MK2 (rhesus monkey epithelial kidney) cells with an immunochemical assay, which reports the percentage of infected cells. In this assay (Table 1, Figure 4), Ala-containing geminoids **2a** and **2b** (C_16_-KA_n_K-C_16_ with *n* = 1 and 2) were much more effective than the Gly-containing **3a** (C_16_-KGK-C_16_), while **3b** (C_16_-KG_2_K-C_16_) was not active. Toxicity was monitored microscopically. Slight toxicity was observed for **2b** (C_16_-KA_2_K-C_16_), **3a** (C_16_-KGK-C_16_, and **3b** (C_16_-KG_2_K-C_16_), but none for **2a** (C_16_-KAK-C_16_). **2a** (C_16_-KAK-C_16_) is, therefore, the most promising compound, even though its IC_50_ and EC_50_ for respectively DENV2 protease inhibition with the Tyr(3-NO_2_) substrate and DENV2 replication are slightly less favourable than those of **2b** (C_16_-KA_2_K-C_16_).

## 3. Discussion

We have found that the geminoids (gemini-like peptide amphiphiles) with two lysines separated by one or two Ala residues (**2a**–**b**) are strong inhibitors, with some IC_50_ values below micromolar, of serine proteases involved in the maturation of DENV capsids, DENV2 protease and furin, with resp. Z-RR-MCA and Ac-RVRR-MCA as the substrates (Table 1). Further studies showed that geminoids of this type inhibit DENV2 replication and viral infection in cultured cells. It is of interest to consider the effect of the amino acids between the linkers; the substitution of hydrogen for a methyl group going from Gly to Ala makes the head group more hydrophobic but also introduces more conformational rigidity, which is reflected in the preferences of the amino acids to be found in certain secondary protein structure elements, where Gly is mostly found in β-turns and Ala in α-helices. The higher polarity of the headgroup in the Gly-containing geminoids might result in less effective cell penetration, which might explain the relatively poor performance of **3a** in the cell infection assay, whereas the rigidity of the Ala-containing geminoids **2** probably favours efficient recognition by the substrate-binding site of DENV2 protease. It should be noted, however, that in the in vitro assays of DENV2 protease, the FRET substrates gave similar results for the geminoid inhibitors but different relative efficacies (Table 1). With Z-RR-MCA, **2a** (C_16_-KAK-C_16_) was a better inhibitor than **2b** (C_16_-KA_2_K-C_16_), and the geminoids with Gly **3a** and **3b** were relatively poor, whereas with the Tyr(3-NO_2_) substrate, **2b** (C_16_-KA_2_K-C_16_) was the best inhibitor, and **3a** (C_16_-KGK-C_16_) was more effective than **2a** (C_16_-KAK-C_16_). This difference may be related to the interaction of the DENV2 protease NS2B and NS3 domains around the active site. NS3 alone is active on relatively small substrates such as Z-RR-MCA, whereas association with NS2B is required for the recognition of larger peptide substrates [8]. The DENV2 protease used in this study is a construct [6,23] in which the NS2B and NS3 fragments are connected by a flexible [39] GGGGSGGGG linker. The DENV2 protease assay with the MCA substrate was carried out in the presence of glycerol, required to stabilise the enzyme in an aqueous solution [40], whereas the buffer for the assay with the Tyr(3-NO_2_) substrate contained ethylene glycol and the non-ionic detergent Brij^®^58 (polyoxyethylene (20) cetyl ether). Importantly, DENV2 protease inhibition by geminoids persists under the latter conditions, which have been designed to suppress the inhibition by non-selective inhibitors [36]. The hydrophobic character of the geminoids promotes the formation of nanoparticles in an aqueous environment, but their CMCs are in the high micromolar range, i.e., considerably higher than the IC_50_ for protease inhibition in vitro. In the attempts to determine the IC_50_ for the inhibition of furin by the Gly geminoids **3**, we observed that the dependence of residual protease activity on inhibitor concentration in vitro showed a disproportional decrease above a concentration of approx. 12 μM (Figure S4). This is quite close to the CMC, at 30 μM for **3b** (C_16_-KG_2_K-C_16_), which is the lowest value found for the selected compounds (Table 1). The enzyme assays with their variety of solutes, buffer salts, glycerol, ethylene glycol, or detergent, are not designed for micelle forming inhibitors. In cells, however, the geminoids are more likely associated with the lipid phase of the membranes than with micelles. No evidence of non-linearity was observed in the concentration range for the experiments in cellular models (Figure 3 and Figure 4), and inhibition of both furin and DENV2 protease was evident at concentrations considerably below the CMC (Table 1. The CMC for **3b** (C_16_-KG_2_K-C_16_, 30 μM) is too high to correspond to either of the transitions in Figure S4 at 1 and 12 μM. Thus, despite the apparent hydrophobic attraction between the molecules of the amphiphilic inhibitors in water, the transitions in Figure S4 cannot be explained by their aggregation alone. In addition to the recognition of the peptide sequence in the enzyme’s active site, hydrophobic interactions between amphiphilic inhibitor and enzyme probably play a role. The interaction of NS3 and NS2B, which is required for full catalytic activity, has recently been identified as a target for allosteric inhibition of DENV2 and Zika proteases [41,42,43]. Although the interactions between the fragments in the open, inactive, ligand-free (DENV2) [39] and closed, active, ligand-bound (DENV3) [44] enzyme conformations are mainly electrostatic, the common structural feature of the first inhibitors that are recognised as allosteric [41,42,43] is that they contain multiple apolar aromatic groups. It is therefore very likely that the apolar alkyl tails of the geminoid inhibitors play a similar role. The relatively good performance of compound **3a** in the DENV2 protease assay with the larger (i.e., the Tyr(3-NO_2_)) substrate may be explained by the aforementioned expected higher flexibility of this Gly-containing geminoid, allowing it to interact with both the substrate and allosteric sites.

Because of their two alkyl tails, geminoids are likely to interact with biological membranes, allowing efficient access of relatively large polar peptide substrates to the endoplasmic reticulum, where they can be presented to a viral or host enzyme at the membrane surface. We suggest that this is a likely explanation for the efficiency of this novel class of amphiphilic inhibitors of viral maturation. In an earlier study on peptide inhibitors, the positive effect of *N*-acylation on the inhibition of furin in cells was ascribed to the improved access to the intact cell and linked to the affinity of furin for membranes [45]. The amphiphilic nature of this class of inhibitors could have multiple advantages for their application as drugs and for their possible translocation into the cell. It is likely that single molecules or nanoparticle aggregates of the amphiphilic cationic peptides can be taken up by the cell by endocytosis, analogous to what has been proposed for lipoplexes with cationic gemini surfactants and geminoids in transfection [30]. The application of additional functional elements such as selected oligosaccharides and peptides would allow receptor-mediated targeting and cellular trafficking [46,47]. The formation of mixed nanoparticles such as those with PEGylated lipids would allow stabilized and targeted delivery from the blood [48].

In the cellular context of the DENV2 replicon assay (Figure 3) in HeLa cells, most of the geminoid compounds that showed activity in the in vitro protease inhibition assays inhibited viral replication. **2a** (C_16_-KAK-C_16_) proved most effective at low toxicity. Furthermore, the compounds significantly reduced wild-type dengue virus’s replication in LLC-MMK2 cells (Figure 4); the geminoids with Ala **2** were much more effective in these experiments than those with Gly **3** (Table 1). Both the DENV2 protease and host proteases, including furin, are involved in the maturation of the viral polyprotein [6] and are inhibited by C_16_-KA_n_K-C_16_ geminoids **2a** and **2b**. Thus, we cannot exclude that both serine proteases are targeted in the inhibition of the virus replication. Importantly, however, this is achieved without the adverse effects expected upon complete furin inhibition. This is consistent with the recent finding that furin inhibitors inhibit the replication of the hepatitis B virus [49] and highly pathogenic avian influenza virus [50] without apparent toxicity.

The discovery that geminoid molecules, originally designed for polynucleotide delivery, are active protease inhibitors that suppress viral replication in a variety of cells is a starting point for the design of the next generation of geminoids with peptide sequences optimised for the interaction with the active sites of the target proteases, and, if considered necessary, for selectivity of inhibition of various viral proteases over host proteases such as furin. For this approach, advantage can be taken of the available X-ray crystallographic structures of the DENV2 protease construct [39,51] and furin [52,53].

## 4. Materials and Methods

### 4.1. General

Aldehyde functionalized resin (4-(4-Formyl-3-methoxyphenoxy) butyryl AM resin, loading 0.98 mmol/g) was obtained from Novabiochem and amino acids were purchased from Bachem and Novabiochem. All other chemicals were acquired from Fluka, Aldrich and Baker. The chemicals were used as received unless stated otherwise. Polyethylene syringe barrels containing 20-micron porous polyethylene frits were acquired from Supelco. Preparative HPLC was performed on a Shimadzu LC-20A Prominence system (Shimadzu’s Hertogenbosch, The Netherlands) equipped with a Gemini NX-C18 column, 150 × 21.20 mm, particle size 10 μm (Phenomenex, Utrecht, The Netherlands). Mass spectra were recorded on a Thermofinnigan LCQ-ESI-ion trap and high-resolution mass spectra (HR-MS) on a JEOL AccuToF (ESI-MS). The samples were dissolved in methanol. ^1^H-NMR spectra were recorded on a Bruker DMX-300 MHz at room temperature. The samples were dissolved in DMSO-d6 unless indicated otherwise. ^1^H-NMR spectra are written in the following format: chemical shift (multiplicity, number of protons); multiplicities: s = singlet; d = doublet; t = triplet; qu = quintet; m = multiplet; b = a broad peak. The FRET substrates Ac-RVRR-MCA [24], Z-RR-MCA [25], and 2-Abz-Nle-Lys-Arg-Arg-Ser-Tyr(3-NO_2_)-NH_2_ [22] were prepared as described in the references given.

### 4.2. Synthesis

The preparation of alkylated peptides of the geminoid type (Figure 1) with C16-tails has been described elsewhere [26,27]. Details of the preparation and characterization of the new series of geminoids **2** (C_16_-KA_n_K-C_16_, 1 < *n* < 4) are given below (with NMR data, including ^1^H and ^13^C NMR spectra for **2a** and **2b**, Figures S7–S10, in the Supporting Information); the preparations of **1** and **3** (C_16_-KG_n_K-C_16_, 0 < *n* < 4, first mentioned in [26]) are given along with their characterization (including ^1^H and ^13^C NMR spectra for **3a** and **3b**, Figures S11–S14 in the Supporting Information).

#### 4.2.1. Synthesis of 2, C_15_H_31_C(O)-Lys-(Ala)n-Lys-NHC_16_H_33_.2TFA (C_16_-KA_n_K-C_16_) for *n* = 1–4

A reductive amination of 1.0092 g aldehyde resin (1.0 mmol) was performed as described elsewhere [26] using 2.4025 g (9.4 mmol) palmitylamine, 694 mg (11 mmol) NaCNBH_3_, and 600 μL AcOH in 30 mL of a 1:1 (*v*/*v*) mixture of DMF/MeOH. The resin was transferred to a syringe marked A and Fmoc-Lys(Boc)-OH was coupled to it using 1.3740 g (2.9 mmol) Fmoc-Lys(Boc)-OH, 3.60 mL 1 M HOBt/DMF (3.60 mmol), and 3.21 mL 1 M DIPCDI/DMF (3.21 mmol). A chloranil test was found to be negative. The resin was subsequently capped using 10 equiv. of acetic acid anhydride and 12 equiv. of pyridine. To the content of syringe (A) Fmoc-Ala-OH (1.0 g, 3.2 mmol) was coupled. Subsequently, from syringe (A) one fourth of the resin was placed into a new syringe (B). Subsequently, Fmoc-Ala-OH (734.3 mg, 2.4 mmol) was coupled to it to the content of syringe (A) and Fmoc-Lys(Boc)-OH (360.0 mg, 0.75 mmol) was coupled to the content of syringe (B). From syringe (A) one third of the resin was placed into a new syringe (C). Subsequently, Fmoc-Ala-OH (494 mg, 1.6 mmol) was coupled to the content of syringe (A), Fmoc-Lys(Boc)-OH (352.8 mg, 0.75 mmol) was coupled to the content of syringe (C), and palmitic acid (195 mg, 0.8 mmol) was coupled to the content of syringe (B). From syringe (A) one half of the resin was placed into a new syringe (D). Subsequently, Fmoc-Ala-OH (264.4 mg, 1.0 mmol) was coupled to the content of syringe (A), Fmoc-Lys(Boc)-OH (365.8 mg, 0.8 mmol) was coupled to the content of syringe (D), and palmitic acid (195 mg, 0.8 mmol) was coupled to the content of syringe (C). Subsequently, Fmoc-Lys(Boc)-OH (361.1 mg, 0.8 mmol) was coupled to the content of syringe (A), palmitic acid (197.3 mg, 0.8 mmol) was coupled to the content of syringe (D), and finally palmitic acid (197.3 mg, 0.8 mmol) was coupled to the content of syringe (A). After washing with diethyl ether and drying the products were cleaved from the resins with 5% H_2_O in TFA for 2–3 h. The products with *n* = 1 and 2 were dissolved in methanol and purified using preparative reverse-phase HPLC; for *n* = 3 and 4 this was not possible due to solubility problems. The mobile phase started as water (0.01% TFA) and went in 15 min to 100% acetonitrile (0.01% TFA), which was retained for 5 min. The fractions with product were collected and dried *in vacuo*.

##### C_15_H_31_C(O)-Lys-(Ala)-Lys-NHC_16_H_33_.2TFA (C_16_-KAK-C_16_, Syringe B)

Yield: 207.9 mg (MW = 1035.33, 0.200 mmol); HR-MS (Positive Ion ESI) [M + H]^+^ calculated (C_47_H_95_N_6_O_4_) 807.74148, found 807.74154; [M + Na]^+^ (C_47_H_94_NaN_6_O_4_) 829.7234, found 829.7267; see Figures S7 and S8 for high-resolution ^1^H and ^13^C NMR spectra, respectively.

##### C_15_H_31_C(O)-Lys-(Ala)_2_-Lys-NHC_16_H_33_.2TFA (C_16_-KA_2_K-C_16_, Syringe C)

Yield: 100.7 mg (MW = 1106.41, 0.091 mmol); HR-MS (Positive Ion ESI) [M + Na]^+^ calculated (C_50_H_99_NaN_7_O_5_) 900.76054, found 900.76476; see Figures S9 and S10 for high-resolution ^1^H and ^13^C NMR spectra, respectively.

##### C_15_H_31_C(O)-Lys-(Ala)_3_-Lys-NHC_16_H_33_.2TFA (C_16_-KA_3_K-C_16_, Syringe D)

Yield: 136.9 mg (MW = 1177.49, 0.116 mmol); HR-MS (Positive Ion ESI) [M + H]^+^ calculated (C_53_H_105_N_8_O_6_) 949.81570, found 949.81969; [M + Na]^+^ (C_53_H_104_NaN_8_O_6_) 971.79765, found 971.80016; see Supporting Information for ^1^H NMR (300 MHz, DMSO-d6) data. Because purification by HPLC was not possible due to solubility problems, compound **2c** was not further investigated.

##### C_15_H_31_C(O)-Lys-(Ala)_4_-Lys-NHC_16_H_33_.2TFA (C_16_-KA_4_K-C_16_, Syringe A)

Yield: 210.0 mg (MW = 1248.57, 0.168 mmol); HR-MS (Positive Ion ESI) [M + H]^+^ calculated (C_56_H_110_N_9_O_7_) 1020.85282, found 1020.85505; [M + Na]^+^ (C_56_H_109_NaN_9_O_7_) 1042.83476, found 1042.83850; see Supporting Information for ^1^H NMR (300 MHz, DMSO-d6) data. Because purification by HPLC was not possible due to solubility problems, compound **2d** was not further investigated.

### 4.3. Critical Micelle Concentration (CMC)

Pyrene was used as a probe to study the changes in its fluorescence, in particular the ratio (I_1_/I_3_) of the intensities I_1_ and I_3_ at between 373 and 383 nm, respectively [32,33]. An abrupt change in this ratio with increasing surfactant concentration points to an increase in hydrophobicity of the environment of the probe corresponding to the formation of aggregates. See Supporting Information for details.

### 4.4. Enzyme Expression, Purification, and Assay

DENV2 protease and human furin were expressed and purified as previously described in refs. [23,54]. See Supporting Information for details.

#### 4.4.1. Furin Assay with MCA Substrate

Furin was dissolved at 0.76 nM concentration in 1 mL MES buffer (10 mM), 1 mM CaCl_2_, pH 7.0 at 36.5 °C. The substrate Ac-RVRR-MCA [25] was added at a concentration of 2.35 μM (10 times the K_m_), and the inhibitor was added in increasing concentrations (as increasing volumes of 1.0, 5.0, 10.0, 20.0, and 40.0 μL) from a stock solution of 2 mg in 1 mL DMSO. The residual activity was measured as fluorescence at 460 nm following excitation at 380 nm in a Hitachi F2500 spectrofluorimeter, and plots were fitted using the Grafit^®^ software (Erithracus Software, Horley, Surrey, UK).

#### 4.4.2. DENV2 Protease Assay

The inhibition reported here was studied on an NS2B-NS3 construct derived from dengue serotype 2 (CF40-GGGGSGGGG-NS3) called DENV2 protease in this study.

- With MCA substrate: The assay was carried out and analysed as described above for furin, but with DENV2 protease at 20 nM concentration in 50 mM Tris.HCl, pH 9.0, 20% glycerol, 37 °C, and with 20 μM Z-RR-MCA as the substrate;
- With Tyr(3-NO_2_) substrate: The applied assay protocol was described by [38]. IC_50_ values were determined in CDD Vault [55] using the Levenberg–Marquardt algorithm for fitting a Hill equation to dose-response data [56,57].

#### 4.4.3. Trypsin Assay

The assay was carried out in 100 mM Tris.HCl, 10 mM CaCl_2_, pH 8.0, with 4 nM enzyme and 11.4 µM Z-FR-MCA substrate.

### 4.5. Replicon Assay and Viability Test

The replicon assay was carried out as described earlier [38] using HeLa cells that contain a stably replicating DENV2 replicon expressing a luciferase reporter gene. The amphiphilic inhibitors were added as concentrated solutions in DMSO; the same amount of DMSO was used as the blank experiment, with the viral inhibitor ribavirin as a positive control. Luciferase activity and cell viability were assessed as described previously [38].

### 4.6. DENV2 IPOX Cytoprotection Assay in LLC-MMK2 Cells

#### 4.6.1. Cell Preparation

LLC-MK2 (Monkey Rhesus Kidney cells; CCL-7.1) were passaged in assay medium (EMEM (Lonza Cat No: BESP069F) supplemented with 10% heat-inactivated FCS (Lonza), 2% Pen/strep (Gibco), 2% L-Glutamine (Gibco), 2% Hepes (Lonza), and 1% sodium bicarbonate (Lonza)) prior to use in the antiviral assay. Cells were seeded in 96-well plates (10^5^ cells/well) in assay medium to be exposed 16–24 h later to compounds and viruses. The plates were incubated at 37 °C/5% CO_2_ overnight to allow for cell adherence.

#### 4.6.2. Compound Preparation

Compounds were solubilized in DMSO and evaluated using two-fold serial dilutions (8-points dose-response curves starting at a concentration of 50 µM) in duplicate for the antiviral assays. Compounds were diluted in assay medium at 1× test concentrations. Ribavirin (Sigma Aldrich, Amsterdam, The Netherlands) was evaluated as a positive control compound in the antiviral assays.

#### 4.6.3. Virus Preparation and Cellular Infection

DENV2 New Guinea strain was grown in AP-61 insect cells (in-house cell bank) in complete Leibovitz medium containing 1% pen/strep (Gibco), 1% L-glutamin (Gibco), 0.5% Hepes (Lonza), 0.5% sodium bicarbonate (Lonza), and 10% tryptose phosphate for the production of stock virus pools. On the day of cellular infection, an aliquot of virus was removed from the freezer (−80 °C) and allowed to thaw in water in a biological safety cabinet. Virus was diluted into assay medium (10^4^ TCID_50_), and 100 µL of this was added to each well, resulting in a TCID_50_ of 100. Cells were incubated for 2 h at 37 °C/5% CO_2_ and washed 3 times with blank assay medium. Directly after washing, 100 µL of the compound dilutions were added to each well.

#### 4.6.4. Plate Format

Each plate contained cell control wells (cells only), virus control wells (cells plus virus), duplicate drug toxicity wells per compound (cells plus drug only), as well as duplicate experimental wells (drug plus cells plus virus).

#### 4.6.5. Immunoperoxidase Staining and Toxicity Determination

Virus-infected cells were visualised using a DENV2 immunoperoxidase staining protocol. Two days after infection, cells were inactivated with ethanol 70% for 30 min and washed with PBS. Fixed plates were incubated with PBS containing 0.05% H_2_O_2_ for 20 min at 37 °C and washed again 3 times with PBS. Plates were incubated for 1 h with 50 µL monoclonal anti-DENV-2 NS1 antibody (Millipore; diluted 1:500 in EMEM). Samples were washed once with PBS containing 0.05% Tween20 and twice with PBS only. Secondary polyclonal goat anti-mouse IgG HRP (Dako; diluted 1:2000) was added 50 µL per well and incubated for 1 h at 37 °C in the dark. Following 3 washing steps with PBS, 100 µL AEC (3-amino-9-ethylcarbazole) substrate buffer (containing 0.03% H_2_O_2_, 3% DMF) was added to each well and incubated for 30 min at room temperature in the dark. Bidest water was added after removal of the substrate solution, and all virus-positive cells per well (marked by brown/red staining) were counted under a microscope. Visual scoring of toxicity per well was performed in parallel.

#### 4.6.6. Data Analysis

First, the numbers of infected cells in duplicate wells were averaged. Subsequently, the average of compound plus virus treated wells was normalised against the average of DMSO plus virus treated wells to calculate percentage inhibition. Processed dose-response data were uploaded in CDD Vault, delivering EC_50_ values for each compound. Qualitative toxicity profiles were uploaded in parallel.

## 5. Patents

Patent WO2017217855A1, B. J. Scholte, M. C. Feiters, M. Damen, Title: “Geminoid Lipopeptide Compounds and Their Uses”, filed 17 June 2016 (NL2016987), published 21 December 2017.

## Figures and Tables

**Table 1:** Properties and activities (IC_50_, µM) of the lysine geminoids **1–3**. Assay conditions, DENV2 protease (MCA): 50 mM Tris.HCl, pH 9.0, 20% glycerol, with 20 nM DENV2 protease and 20 mM Z-RR-MCA, 37 °C; furin: 10 mM Mes. NaOH, pH 7.0, with 0.76 nM furin and 2.35 mM Ac-RVRR-MCA, 37 °C; trypsin: 100 mM Tris.HCl, pH 8.0, 10 mM CaCl_2_, with 4 nM trypsin and 11.4 μM Z-FR-MCA; DENV2 with 50 μM concentration Tyr(3-NO_2_) substrate in 50 mM Tris.HCl, pH 9.0, ethylene glycol (10% *v*/*v*), Brij^®^58 (0.0016%).

Structure	Compound	IC_50_ (µM) (MCA Substrates)			IC_50_ (µM) Tyr(3-NO_2_)	CMC (µM) ^(a)^	DENV Replication in LLC-MK2 Cells	
		DENV2	Furin	Trypsin	DENV2		EC_50_ (µM)	Toxicity
C_16_-KK-C_16_	**1**	4.25 ± 0.27	n.a.	85.7 ± 4.4	n.d.	n.d.	n.d.	n.d.
C_16_-KAK-C_16_	**2a**	0.66 ± 0.07	3.57 ± 0.18	17.18 ± 0.66	2.3 ± 0.7	48–58	4.1 ± 1.5	none
C_16_-KA_2_K-C_16_	**2b**	0.80 ± 0.04	2.14 ± 0.10	20.93 ± 0.34	1.4 ± 0.1	41	3.1 ± 0.7	slight
C_16_-KGK-C_16_	**3a**	1.94 ± 0.14	^(b)^	41 ± 2	2.1 ± 1.1	55–72	12.7 ± 1.1	slight
C_16_-KG_2_K-C_16_	**3b**	3.69 ± 0.50	^(c)^	n.d.	10.2 ± 1.1	30	n.a.	slight

n.d.: not determined; n.a.: not active. ^(a)^ Critical Micelle Concentration. ^(b)^ IC_50_ not determined. ^(c)^ IC_50_ not determined; see the profiles in Figure S4.

## Data Availability

Not applicable.

## Supplementary Materials

The following supporting information can be downloaded at: https://www.mdpi.com/article/10.3390/molecules27103217/s1, Figure S1: IC_50_ curves (values in inset in µM) of inhibition of DENV2 protease with substrate Z-RR-MCA. Figure S2: IC_50_ curves (values in inset in µM) of inhibition of furin by compounds **2** with substrate Ac-RVRR-MCA. Figure S3: IC_50_ curves (values in inset in µM) of inhibition of trypsin by selected compounds with substrate Z-FR-MCA. Figure S4: Inhibition of furin by **3b** with substrate Ac-RVRR-MCA. Figure S5: Determination of the critical micelle concentration (CMC). Procedures: Enzyme expression and purification. Figure S6: Toxicity of the compounds tested with Celltiter Blue Viability Assay (Promega) using HeLa cells (wildtype). Synthesis and characterization (including subjective assignments) of **2** (C_15_H_31_C(O)-Lys-(Ala)_n_-Lys-NHC_16_H_33_.2TFA, C_16_-KA_n_K-C_16_ with *n* = 1–4) and **1** and **3** (C_15_H_31_C(O)-Lys-(Gly)_n_-Lys-NHC_16_H_33_.2TFA, C_16_-KG_n_K-C_16_ with *n* = 0–4). Figure S7: ^1^H NMR of **2a** (C_16_-KAK-C_16_). Figure S8: ^13^C NMR of **2a** (C_16_-KAK-C_16_). Figure S9: ^1^H NMR of **2b** (C_16_-KA_2_K-C_16_). Figure S10: ^13^C NMR of **2b** (C_16_-KA_2_K-C_16_). Figure S11: ^1^H NMR of **3a** (C_16_-KGK-C_16_). Figure S12: ^13^C NMR of **3a** (C_16_-KGK-C_16_). Figure S13: ^1^H NMR of **3b** (C_16_-KG_2_K-C_16_). Figure S14. ^13^C NMR of **3b** (C_16_-KG_2_K-C_16_).

## Sample Availability

Samples of the compounds are not available from the authors.
