# Supplementary material for: Cationic Geminoid Peptide Amphiphiles Inhibit DENV2 Protease, Furin, and Viral Replication
Source: Molecules. 2022 May 17;27(10):3217. doi: 10.3390/molecules27103217 (PMC9143577; doi:10.3390/molecules27103217)
Supplement: Supplementary file 1 [file molecules-27-03217-s001.zip › molecules-1711817-supplementary.pdf]

Supporting Information for the paper Molecules 1711817

'Cationic geminoid peptide amphiphiles inhibit DENV2 protease, furin, and viral replication'

by Mark Damen, Mario A. Izidoro, Debora N. Okamoto, Lilian C. G. Oliveira, Helene I. V. Amatdjais-Groenen, Stijn F. M. van Dongen, Koen W. R. van Cleef, Ronald P. van Rij, Cindy E. J. Dieteren, Daniel Gironés, Bernd N. M. van Buuren, Byron E. E. Martina, Albert D. M. E. Osterhaus, Luiz Juliano, Bob J. Scholte, and Martin C. Feiters

Figure S1. IC_{50} curves (values in inset in μM) of inhibition of DENV2 protease with substrate Z-RR-MCA.

Figure S2. IC_{50} curves (values in inset in μM) of inhibition of furin by compounds **2** with substrate Ac-RVRR-MCA.

Figure S3. IC_{50} curves (values in inset in μM) of inhibition of trypsin by selected compounds with substrate Z-FR-MCA.

Figure S4. Inhibition of furin by **3b** with substrate Ac-RVRR-MCA.

Figure S5. Determination of the critical micelle concentration (CMC)

Enzyme expression and purification

Figure S6. Toxicity of the compounds tested with Celltiter Blue Viability Assay (Promega) using HeLa cells (wildtype).

Synthesis and characterization (including subjective assignments) of **2** ($C_{15}H_{31}C(O)$-Lys-$(Ala)_n$-Lys-$NHC_{16}H_{33}$.2TFA, C_{16}-KA_nK-C_{16} with n=1-4) and **1** and **3** ($C_{15}H_{31}C(O)$-Lys-$(Gly)_n$-Lys-$NHC_{16}H_{33}$.2TFA,C_{16}-KG_nK-C_{16} with n=0-4)

Figure S7. ^{1}H NMR of **2a** (C_{16}-KAK-C_{16})

Figure S8. ^{13}C NMR of **2a** (C_{16}-KAK-C_{16})

Figure S9. ^{1}H NMR of **2b** (C_{16}-KA_2K-C_{16})

Figure S10. ^{13}C NMR of **2b** (C_{16}-KA_2K-C_{16})

Figure S11. ^{1}H NMR of **3a** (C_{16}-KGK-C_{16})

Figure S12. ^{13}C NMR of **3a** (C_{16}-KGK-C_{16})

Figure S13. ^{1}H NMR of **3b** (C_{16}-KG_2K-C_{16})

Figure S14. ^{13}C NMR of **3b** (C_{16}-KG_2K-C_{16})

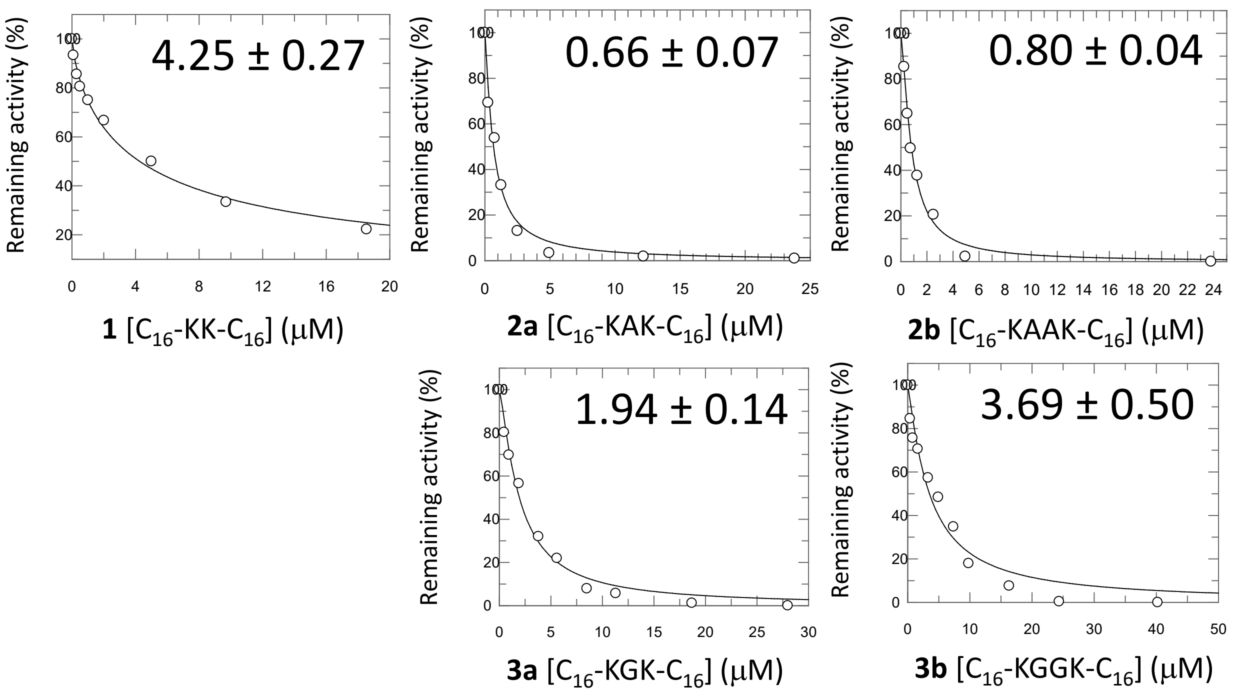

Figure S1. IC_{50} curves (values in inset in μM) of inhibition of DENV2 protease with substrate Z-RR-MCA. Retained activity, fluorescence (arbitrary units)/min normalized to 100 % for 0 μM inhibitor concentration.

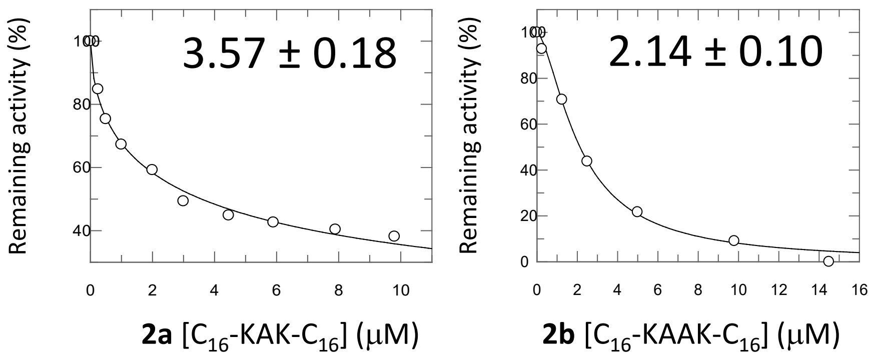

Figure S2. IC_{50} curves (values in inset in μM) of inhibition of furin by compounds **2** with substrate Ac-RVRR-MCA. Retained activity, fluorescence (arbitrary units)/min normalized to 100 % for 0 μM inhibitor concentration.

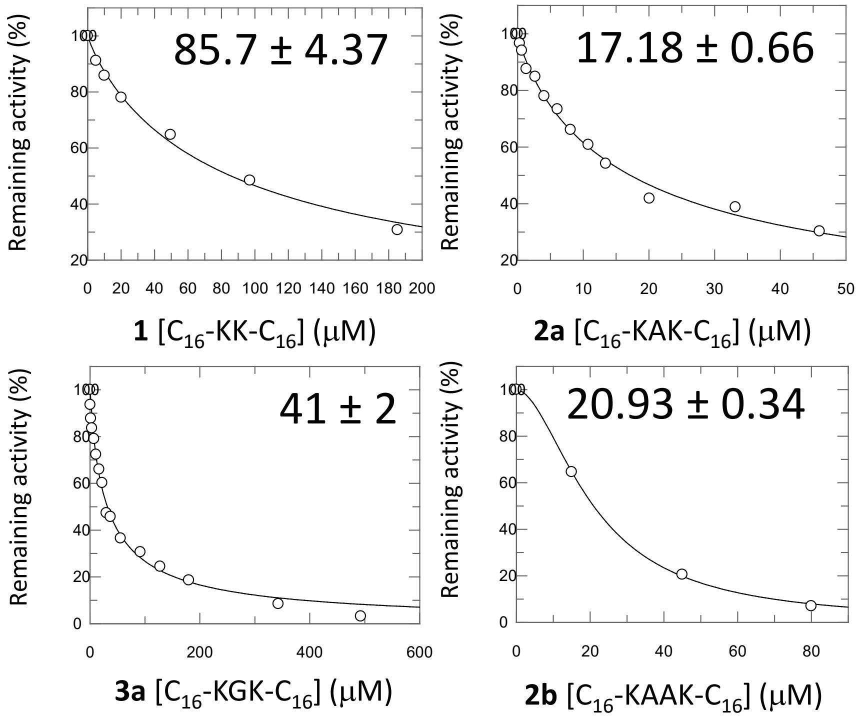

Figure S3. IC_{50} curves (values in inset in μM) of inhibition of trypsin by selected compounds with substrate Z-FR-MCA. Retained activity, fluorescence (arbitrary units)/min normalized to 100 % for 0 μM inhibitor concentration.

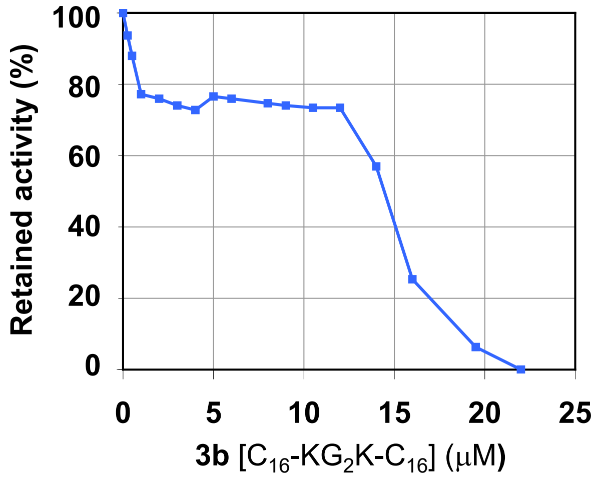

Figure S4. Inhibition of furin by **3b** with substrate Ac-RVRR-MCA. Retained activity, fluorescence (arbitrary units)/min normalized to 100 % for 0 μM inhibitor concentration.

Determination of the critical micelle concentration (CMC)

A solution of 5 mg pyrene in methanol (10 mL) was prepared and diluted 20 times. 50 μL from the diluted solution was added to geminoid solutions in water to a final volume of 3 mL. The geminoid samples were made from a stock solution of 500 μM in water in a concentration range from 5 to 200 μM. The fluorescence spectra were then measured after 1 hour with the extinction set to 334 nm and emission range 350-500 nm, excitation slit 8.0 mm, emission slit 2.5 mm and at 25 °C. The ratio of the intensities I_I/I_{III} was then plotted against the geminoid concentration, and the CMC is calculated from the intersection point of the best fits to the data points below and above it (Figure S5).

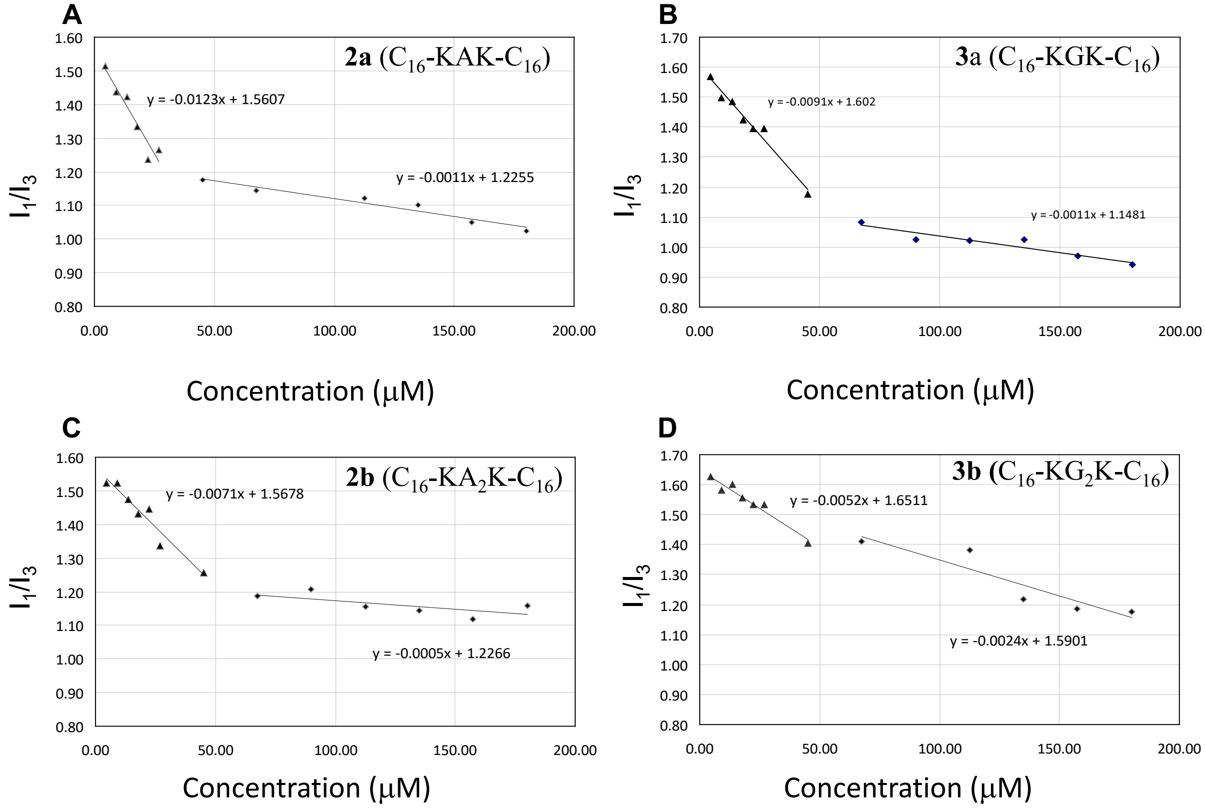

Figure S5. Determination of the critical micelle concentration (CMC) of A) **2a** (C_{16}-KAK-C_{16}, y = -0.0052x + 1.6511 = -0.0024x + 1.5901; x = 44 μM), B) **2b** (C_{16}-KA_2K-C_{16}, y = -0.0071x + 1.5678 = -0.0005x + 1.2266; x = 52 μM), C) **3a** (C_{16}-KGK-C_{16}, y = -0.0091x + 1.602 = -0.0011x + 1.1481; x = 57 μM), and D) **3b** (C_{16}-KG_2K-C_{16}, y = -0.0123x + 1.5607 = -0.0011x + 1.2255; x = 30 μM).

Enzyme expression and purification

DENV2 Protease Preparation. The enzyme was expressed and purified as previously described (ref. S1). Briefly, the vector with CF40 fused to NS3pro was used for high-level inducible expression of recombinant enzyme, tagged on the N-terminus with hexahistidine. *E. coli* strain SG13009 cells were transformed with the expression plasmid and grown in kanamycin at 37 ºC until the absorbance at 600 nm reached a value of 0.6. Cells were induced for expression by the addition of isopropyl-β-D-thiogalactopyranose to a final concentration of 1 mM and incubated for additional 3 h at 30 ºC. The cells were harvested by centrifugation, resuspended in 1 mL of a lysis buffer, subjected to sonication, and centrifuged again. The supernatant was collected and passed through a 2-mL column of nitrilotriacetic acid-agarose (Qiagen) loaded with Ni^{2+}. The column was extensively washed with buffer containing 100 mM imidazole, and the protein was then eluted from then column in buffer containing 100 mM imidazole. Elution samples were analyzed by 15% SDS-PAGE and the substrate Z-RR-MCA was used to distinguish the activity of DENV2 protease from that of contaminating bacterial proteases. The molar concentration was determined by active-site titration with aprotinin (ref. S2).

Human Furin Preparation. The enzyme was expressed and purified as previously described (ref. S3). Briefly, The PCMV-Fur_S vector containing cDNA encoding truncated human furin was obtained from Ayoubi et al. (ref. S4). CHO DG44 cells were stably transfected and expression-amplified using the dihydrofolate reductase-coupled amplification method as described elsewhere (ref. S5). Human furin was purified following the method described for mouse furin purification (ref. S6). Its molar concentration of the active enzyme was determined by active site titration using the decanoyl-RVKR-CMK inhibitor (ref. S7) in 10 mM MES, 1 mM $CaCl_2$, pH 7.0 and the substrate Abz-GIRRKRSVSHQ-EDDnp.

Celltiter Blue Viability Assay for 2a-b and 3

The toxicity of the compounds **2a-b** (C_{16}-KA_nK-C_{16} with n = 1 or 2) and **3a-b** (C_{16}-KG_nK-C_{16} with n = 1 or 2) was tested with Celltiter Blue Viability Assay (Promega) using HeLa cells (wildtype). Cells were incubated in 96 well plates for 24 h in presence of the compounds. A concentration range from 0.8 to 50 µM was used. No CC_{50} could be calculated at this concentration range. Only **3b** (in one experiment) showed slight toxicity at the highest concentrations (Figure S6):

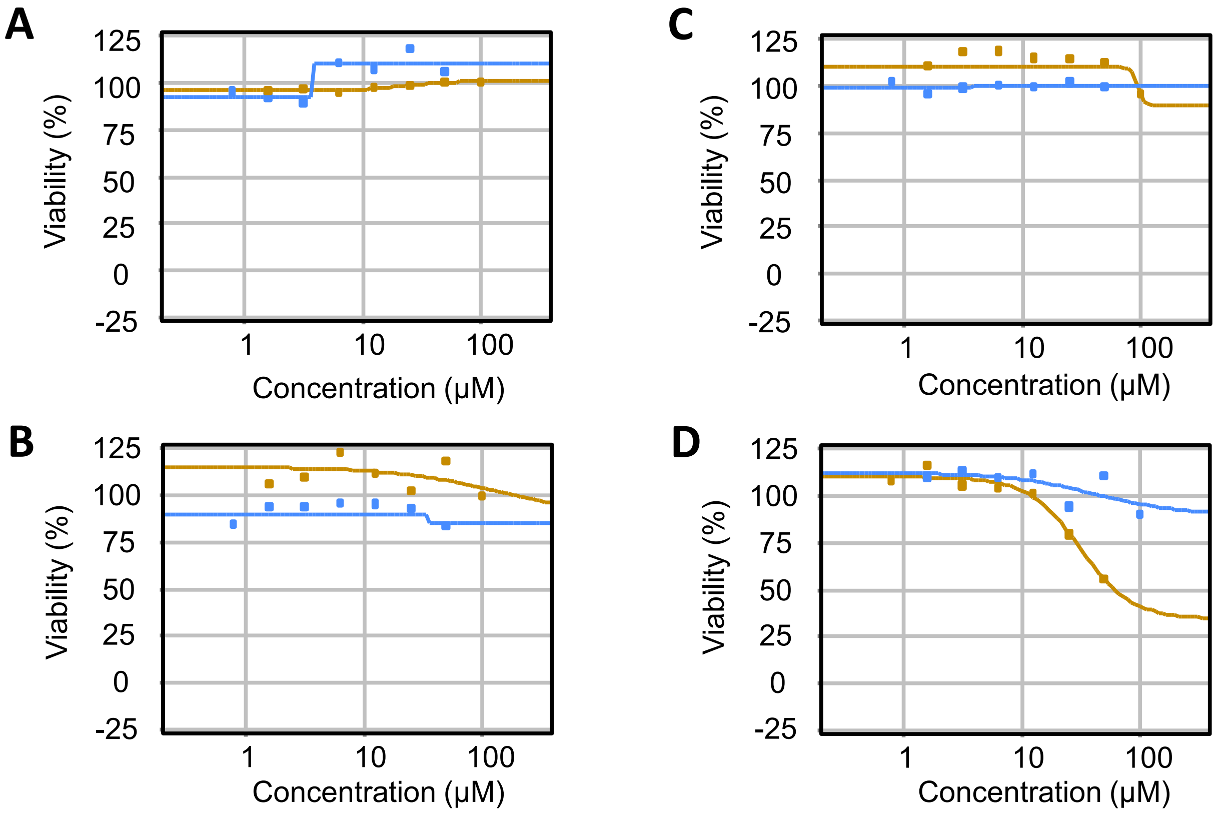

Figure S6. **Toxicity of the compounds** was tested with Celltiter Blue Viability Assay (Promega) using HeLa cells (wildtype). The panels show the average curves (drawn by CDD vault) of two different experiments performed *in triplicate*. Only compound **3b** (C_{16}-KG_2K-C_{16}) shows some cytotoxicity at concentrations >25 µM, but it is not sufficient for CC_{50} calculation.

Synthesis of 2, $C_{15}H_{31}C(O)$-Lys-(Ala)$_n$-Lys-$NHC_{16}H_{33}$.2TFA (C_{16}-KA$_n$K-C_{16}) with n = 1-4. A reductive amination of 1.0092 g aldehyde resin (1.0 mmol) was performed as described elsewhere (ref. S8) using 2.4025 g (9.4 mmol) palmitylamine, 694 mg (11 mmol) $NaCNBH_3$, and 600 μL AcOH in 30 mL of a 1:1 (v/v) mixture of DMF/MeOH. The resin was transferred to a syringe marked A and Fmoc-Lys(Boc)-OH was coupled to it using 1.3740 g (2.9 mmol) Fmoc-Lys(Boc)-OH, 3.60 mL 1 M HOBt/DMF (3.60 mmol), and 3.21 mL 1 M DIPCDI/DMF (3.21 mmol). A chloranil test was found to be negative. The resin was subsequently capped using 10 equiv. of acetic acid anhydride and 12 equiv. of pyridine. To the content of syringe (A) Fmoc-Ala-OH (1.0 g, 3.2 mmol) was coupled. Subsequently, from syringe (A) one fourth of the resin was placed into a new syringe (B). Subsequently, Fmoc-Ala-OH (734.3 mg, 2.4 mmol) was coupled to it to the content of syringe (A) and Fmoc-Lys(Boc)-OH (360.0 mg, 0.75 mmol) was coupled to the content of syringe (B). From syringe (A) one third of the resin was placed into a new syringe (C). Subsequently, Fmoc-Ala-OH (494 mg, 1.6 mmol) was coupled to the content of syringe (A), Fmoc-Lys(Boc)-OH (352.8 mg. 0.75 mmol) was coupled to the content of syringe (C), and palmitic acid (195 mg, 0.8 mmol) was coupled to the content of syringe (B). From syringe (A) one half of the resin was placed into a new syringe (D). Subsequently, Fmoc-Ala-OH (264.4 mg, 1.0 mmol) was coupled to the content of syringe (A), Fmoc-Lys(Boc)-OH (365.8 mg. 0.8 mmol) was coupled to the content of syringe (D), and palmitic acid (195 mg, 0.8 mmol) was coupled to the content of syringe (C). Subsequently, Fmoc-Lys(Boc)-OH (361.1 mg. 0.8 mmol) was coupled to the content of syringe (A), palmitic acid (197.3 mg, 0.8 mmol) was coupled to the content of syringe (D), and finally palmitic acid (197.3 mg, 0.8 mmol) was coupled to the content of syringe (A). After washing with diethyl ether and drying the products were cleaved from the resins with 5% H_2O in TFA for 2–3 h. The products **2a** and **2b** (n = 1 resp. 2) were dissolved in methanol and purified using preparative reverse-phase HPLC; for **2c** and **2d** (n = 3 resp. 4) this was not possible due to solubility problems. The mobile phase started as water (0.01% TFA) and went in 15 minutes to 100% acetonitrile (0.01% TFA) which was retained for 5 minutes. The fractions with product were collected and dried *in vacuo*.

2a, $C_{15}H_{31}C(O)$-Lys-(Ala)-Lys-$NHC_{16}H_{33}$.2TFA (C_{16}-KAK-C_{16}, syringe B): Yield: 207.9 mg (MW = 1035.33, 0.200 mmol); HR-MS (Positive Ion ESI) $[M+H]^+$ calculated ($C_{47}H_{95}N_6O_4$) 807.74148, found 807.74154, $[M+Na]^+$ ($C_{47}H_{94}NaN_6O_4$) 829.7234, found 829.7267; See Figures S7 and S8 for ^1H NMR and ^{13}C NMR.

2b, $C_{15}H_{31}C(O)$-Lys-(Ala)$_2$-Lys-$NHC_{16}H_{33}$.2TFA (C_{16}-KA$_2$K-C_{16}, syringe C): Yield: 100.7 mg (MW = 1106.41, 0.091 mmol); HR-MS (Positive Ion ESI) $[M+Na]^+$ calculated ($C_{50}H_{99}NaN_7O_5$) 900.76054, found 900.76476; See Figures S9 and S10 for ^1H NMR and ^{13}C NMR.

2c, $C_{15}H_{31}C(O)$-Lys-(Ala)$_3$-Lys-$NHC_{16}H_{33}$.2TFA (C_{16}-KA$_3$K-C_{16}, syringe D): Yield: 136.9 mg (MW = 1177.49, 0.116 mmol); HR-MS (Positive Ion ESI) $[M+H]^+$ calculated ($C_{53}H_{105}N_8O_6$) 949.81570, found 949.81969, $[M+Na]^+$ ($C_{53}H_{104}NaN_8O_6$) 971.79765, found 971.80016; ^1H NMR (300 MHz, DMSO-d6) δ: 7.78 (d, NH), 7.51 (d, NH), 7.98 (d, NH), 7.24 (m, NH), 4.31 (m, 3H, AA:H*), 3.63 (m, n.a.), 2.99 (t, 2H, C_{16}:CH_2NHC=O),

2.72 (t, 4H, Lys:C$H_2$$NH_2$), 2.07 (m, 2H, C_{16}:CH_2C=O), 1.60 (m, 4H, Lys: β-CH_2), 1.48 (br, 3H, Ala:CH_3 & 6H, Lys/C_{16}:C$H_2$$CH_2$NC=O & 2H, C_{16}:C$H_2$$CH_2$C=O), 1.21 (br, 50H, Lys/C_{16}:CH_2) 0.83 (t, 6H, C_{16}:CH_3). Because purification by HPLC was not possible due to solubility problems, compound **2c** was not further investigated.

2d, $C_{15}H_{31}$C(O)-Lys-(Ala)$_4$-Lys-NH$C_{16}H_{33}$.2TFA (C_{16}-KA_4K-C_{16}, syringe A): Yield: 210.0 mg (MW = 1248.57, 0.168 mmol); HR-MS (Positive Ion ESI) $[M+H]^+$ calculated ($C_{56}H_{110}N_9O_7$) 1020.85282, found 1020.85505, $[M+Na]^+$ ($C_{56}H_{109}NaN_9O_7$) 1042.83476, found 1042.83850 ; ^{1}H NMR (300 MHz, DMSO-d6) δ: 7.97 (d, NH), 7.51 (d, NH), 7.69 (d, NH), 7.46 (m, NH), 7.14 (m, NH), 4.18 (m, 3H, AA:H*), 3.63 (m, n.a.), 2.97 (m, 2H, C_{16}:CH_2NHC=O), 2.70 (m, 4H, Lys:C$H_2$$NH_2$), 2.08 (m, 2H, C_{16}:CH_2C=O), 1.60 (m, 4H, Lys: β-CH_2), 1.47 (br, 3H, Ala:CH_3 & 6H, Lys/C_{16}:C$H_2$$CH_2$NC=O & 2H, C_{16}:C$H_2$$CH_2$C=O), 1.21 (br, 50H, Lys/C_{16}:CH_2) 0.83 (t, 6H, C_{16}:CH_3). Because purification by HPLC was not possible due to solubility problems, compound **2d** was not further investigated.

Synthesis of 1 and 3, $C_{15}H_{31}$C(O)-Lys-(Gly)$_n$-Lys-NH$C_{16}H_{33}$.2TFA (C_{16}-KG_nK-C_{16}) with n = 0-4. A reductive amination of 1.0 g aldehyde resin (1.0 mmol) was performed as described elsewhere (ref. S8) using 2.1729 g palmitylamine (9 mmol), 713.6 mg $NaCNBH_3$ (11 mmol), and 600 µL AcOH in 90 mL of a 1:1 (v/v) mixture of DMF/MeOH. The resin was transferred to a syringe marked A and Fmoc-Lys(Boc)-OH was coupled to it two times using 1.3757 g (2.9 mmol) Fmoc-Lys(Boc)-OH, 3.60 mL 1 M HOBt/DMF (3.60 mmol) and 3.21 mL 1 M DIPCDI/DMF (3.21 mmol) as described in section 4.1-4.4. A chloranil test was found to be negative. The resin was subsequently capped. From syringe A, one fifth of the resin was placed into a new syringe (B). To the content of syringe (A) Fmoc-Gly-OH (963.0 mg, 3.2 mmol) was coupled and to that of syringe (B) Fmoc-Lys(Boc)-OH (377 mg, 0.8 mmol). Subsequently, from syringe (A) one fourth of the resin was placed into a new syringe (C). Thereafter, Fmoc-Gly-OH (721 mg, 2.4 mmol) was coupled to the content of syringe (A), Fmoc-Lys(Boc)-OH (354 mg, 0.8 mmol) was coupled to the content of syringe (C), and palmitic acid (521.9 mg, 2 mmol) was coupled to the content of syringe (B). From syringe (A) one third of the resin was placed into a new syringe (D). Subsequently, Fmoc-Gly-OH (479 mg, 1.6 mmol) was coupled to the content of syringe (A), Fmoc-Lys(Boc)-OH (349 mg, 0.7 mmol) was coupled to the content of syringe (D), and palmitic acid (419 mg, 1.6 mmol) was coupled to the content of syringe (C). From syringe (A) half of the resin was placed into a new syringe (E). Thereafter, Fmoc-Gly-OH (300 mg, 1.0 mmol) was coupled to the content of syringe (A), Fmoc-Lys(Boc)-OH (368 mg. 0.8 mmol) was coupled to the content of syringe (E), and palmitic acid (494 g, 1.9 mmol) was coupled to the content of syringe (D). Subsequently, Fmoc-Lys(Boc)-OH (523 mg. 0.8 mmol) was coupled to the content of syringe (E), palmitic acid (523 mg, 2.0 mmol) was coupled to the content of syringe (D), and finally palmitic acid (523 mg, 2.0 mmol) was coupled to the content of syringe (A). After washing with diethyl ether and drying the products were cleaved from the resins. The products were dissolved in methanol and purified using preparative reverse-phase HPLC. The mobile phase started as water (0.01% TFA) and went in 15 minutes to 100% acetonitrile (0.01% TFA) which was retained for 5 minutes. The fractions with

product were collected and dried *in vacuo*. Purification by preparative reverse-phase HPLC was not possible for **3c** and **3d** (n = 3 resp. 4) due to solubility problems.

Resin loading was determined to be 0.6746 mmol/g.

1, $C_{15}H_{31}C(O)$-Lys-Lys-$NHC_{16}H_{33}$.2TFA (C_{16}-KK-C_{16}, syringe B): Yield: nd (MW = 964.24); MALDI-TOF (DHB) calculated: 735.70 ($C_{44}H_{89}N_5O_3$), found: 758.86 $[M+Na]^+$; ^1H NMR (300 MHz, DMSO-d6) δ: 7.95 – 7.67 (m, 3H, NH), 4.14 (m, 2H, Lys:H^*), 2.97 (m, 2H, C_{16}:CH_2NH), 2.70 (m, 4H, Lys:CH_2NH), 2.25 (m, 2H, C_{16}:CH_2C=O), 2.04 (m, 4H, Lys:NH_2), 1.63 (m, 4H, Lys:CH_2CH), 1.47 (m, 2H, C_{16}:C$H_2$$CH_2$C=O & 6H, C_{16}/Lys:C$H_2$$CH_2$NH), 1.21 (br, 54H, Lys/$C_{16}$:$CH_2$), 0.83 (t, 6H, C_{16}:CH_3).

3a, $C_{15}H_{31}C(O)$-Lys-(Gly)-Lys-$NHC_{16}H_{33}$.2TFA (C_{16}-KGK-C_{16}, syringe C)**:** Yield: nd (MW = 1021.31); HR-MS (Positive Ion ESI) $[M+H]^+$ calculated ($C_{46}H_{93}N_6O_4$) 793.72583, found 793.72625, $[M+Na]^+$ ($C_{46}H_{92}NaN_6O_4$) 815.70777, found 815.70875; See Figures S11 and S12 for ^1H NMR and ^{13}C NMR.

3b, $C_{15}H_{31}C(O)$-Lys-$(Gly)_2$-Lys-$NHC_{16}H_{33}$.2TFA (C_{16}-KG_2K-C_{16}, syringe D)**:** Yield: nd (MW = 1078.36); HR-MS (Positive Ion ESI) $[M+H]^+$ calculated ($C_{48}H_{96}N_7O_5$) 850.74729, found 850.74713, $[M+Na]^+$ ($C_{48}H_{95}NaN_7O_5$) 872.72924, found 872.73161; See Figures S13 and S14 for ^1H NMR and ^{13}C NMR.

3c, $C_{15}H_{31}C(O)$-Lys-$(Gly)_3$-Lys-$NHC_{16}H_{33}$.2TFA (C_{16}-KG_3K-C_{16}, syringe E)**:** Yield: nd (MW = 1135.41); HR-MS (Positive Ion ESI) $[M+H]^+$ calculated ($C_{50}H_{99}N_8O_6$) 907.76875, found 907.77120; ^1H NMR (300MHz, DMSO-d6) δ: 8.15 – 7.68 (m, 6H, NH), 4.18 (m, 2H, Lys:H^*), 3.72 (m, 6H, Gly:CH_2), 2.99 (m, 2H, C_{16}:CH_2NH), 2.71 (m, 4H, Lys:CH_2NH), 2.25 (m, 2H, C_{16}:CH_2C=O), 2.11 (m, 4H, Lys:NH_2), 1.63 (m, 4H, Lys:CH_2CH), 1.47 (m, 2H, C_{16}:C$H_2$$CH_2$C=O & 6H, C_{16}/Lys:C$H_2$$CH_2$NH), 1.21 (br, 54H, Lys/$C_{16}$:$CH_2$), 0.83 (t, 6H, C_{16}:CH_3). Because purification by HPLC was not possible due to solubility problems, compound **3c** was not further investigated.

3d, $C_{15}H_{31}C(O)$-Lys-$(Gly)_4$-Lys-$NHC_{16}H_{33}$.2TFA (C_{16}-KG_4K-C_{16}, syringe A)**:** Yield: nd (MW = 1192.46); HR-MS (Positive Ion ESI) $[M+H]^+$ calculated ($C_{52}H_{102}N_9O_7$) 964.79022, found 964.79380, $[M+Na]^+$ ($C_{52}H_{101}NaN_9O_7$) 986.77216, found 986.77626; ^1H NMR (300 MHz, DMSO-d6) δ: 8.24 – 7.88 (m, 8H, NH), 4.20 (m, 3H, Lys:H^*), 3.74 (br, 8H, Gly:CH_2), 3.00 (m, 2H, C_{16}:CH_2NH), 2.71 (m, 6H, Lys:CH_2NH), 2.29 (m, 2H, C_{16}:CH_2C=O), 2.11 (br, 6H, Lys:NH_2), 1.64 (br, 6H, Lys:CH_2CH), 1.52 (br, 2H, C_{16}:C$H_2$$CH_2$C=O & 8H, C_{16}/Lys:C$H_2$$CH_2$NH), 1.21 (br, 58H, Lys/$C_{16}$:$CH_2$), 0.83 (t, 6H, C_{16}:CH_3). Because purification by HPLC was not possible due to solubility problems, compound **3d** was not further investigated.

Figure S7. ^{1}H NMR of 2a (C_{16}-KAK-C_{16})

^{1}H NMR (400 MHz, DMSO-d6) δ 7.99 (d, *J* = 7.6 Hz, 1H), 7.93 (d, *J* = 6.9 Hz, 1H), 7.83 (d, *J* = 8.2 Hz, 1H), 7.74 (t, *J* = 5.6 Hz, 1H), 7.68 (m, NH_2), 4.21 (d, *J* = 14.3 Hz, 1H), 4.17 – 4.11 (m, 2H), 3.02 (q, *J* = 6.4 Hz, 2H), 2.75 (td, *J* = 7.5, 4.7 Hz, 4H), 2.05 (t, *J* = 7.5 Hz, 2H), 1.70 – 1.60 (m, 2H), 1.45 – 1.55 (m, 8H), 1.42 – 1.31 (m, 2H), 1.23 (s, 52H), 0.89 – 0.81 (m, 6H).

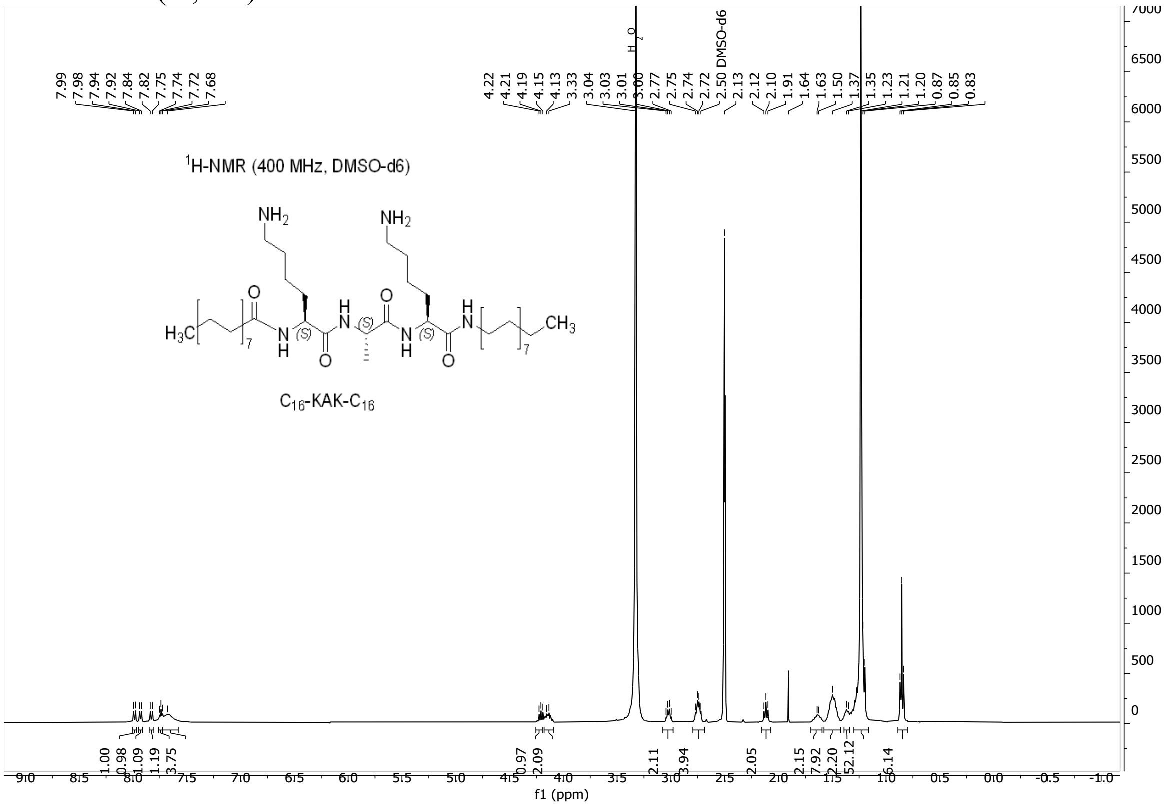

Figure S8. ^{13}C NMR of 2a (C_{16}-KAK-C_{16})

^{13}C NMR (APT mode, 101 MHz, $CDCl_3$/MeOD-d4) δ 175.45, 173.70, 173.42, 172.33, 53.53, 52.97, 50.11, 39.87, 39.44, 36.20, 32.16, 29.93, 29.92, 29.89, 29.85, 29.79, 29.63, 29.59, 29.40, 27.15, 25.93, 22.90, 22.26, 16.87, 14.18.

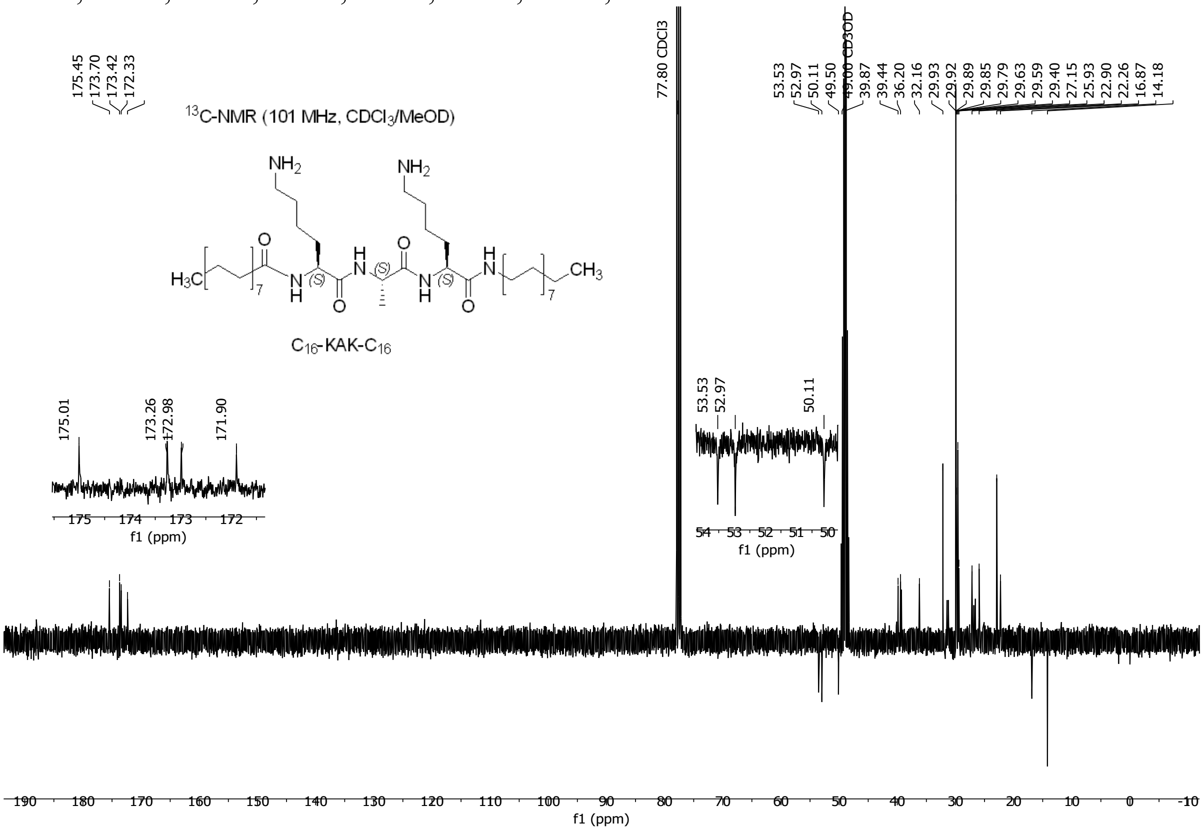

Figure S9. ^{1}H NMR of 2b (C_{16}-KA$_2$K-C_{16})

^{1}H NMR (400 MHz, DMF-d7) δ 4.31 – 4.18 (m, 4H), 3.14 (t, *J* = 6.6 Hz, 2H), 3.04 (dt, *J* = 7.2, 4.7 Hz, 4H), 2.25 (t, *J* = 7.4 Hz, 2H), 1.79 – 1.65 (m, 4H), 1.61 – 1.52 (m, 2H), 1.52 – 1.41 (m, 4H), 1.34 (t, *J* = 7.8 Hz, 4H), 1.26 (s, 40H), 0.90 – 0.81 (m, 6H).

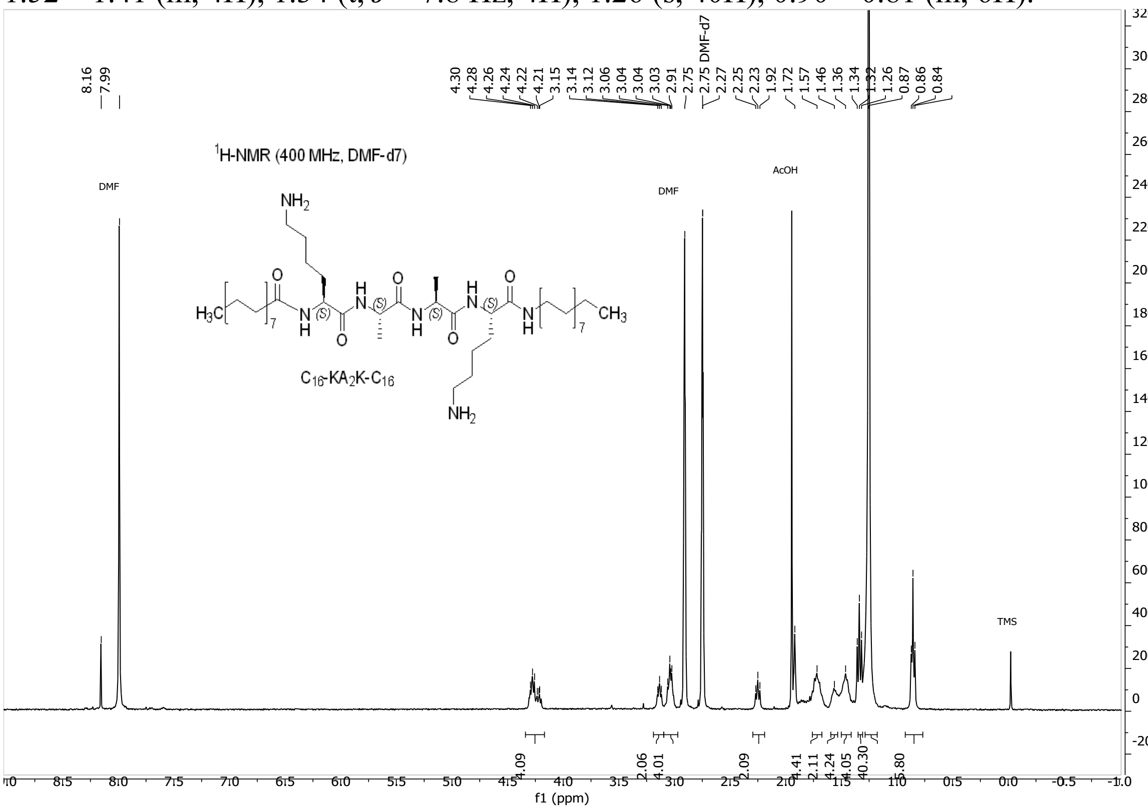

Figure S10. ^{13}C NMR of 2b (C_{16}-KA$_2$K-C_{16})

^{13}C NMR (APT mode, 101 MHz, $CDCl_3$/DMF-d7) δ 172.86, 171.53, 171.06, 170.16, 52.88, 51.53, 48.47, 48.35, 37.94, 37.45, 33.00, 32.79, 30.20, 27.98, 27.96, 27.93, 27.89, 27.75, f27.63, 27.62, 25.28, 25.15, 25.04, 23.90, 20.92, 18.01, 12.13.

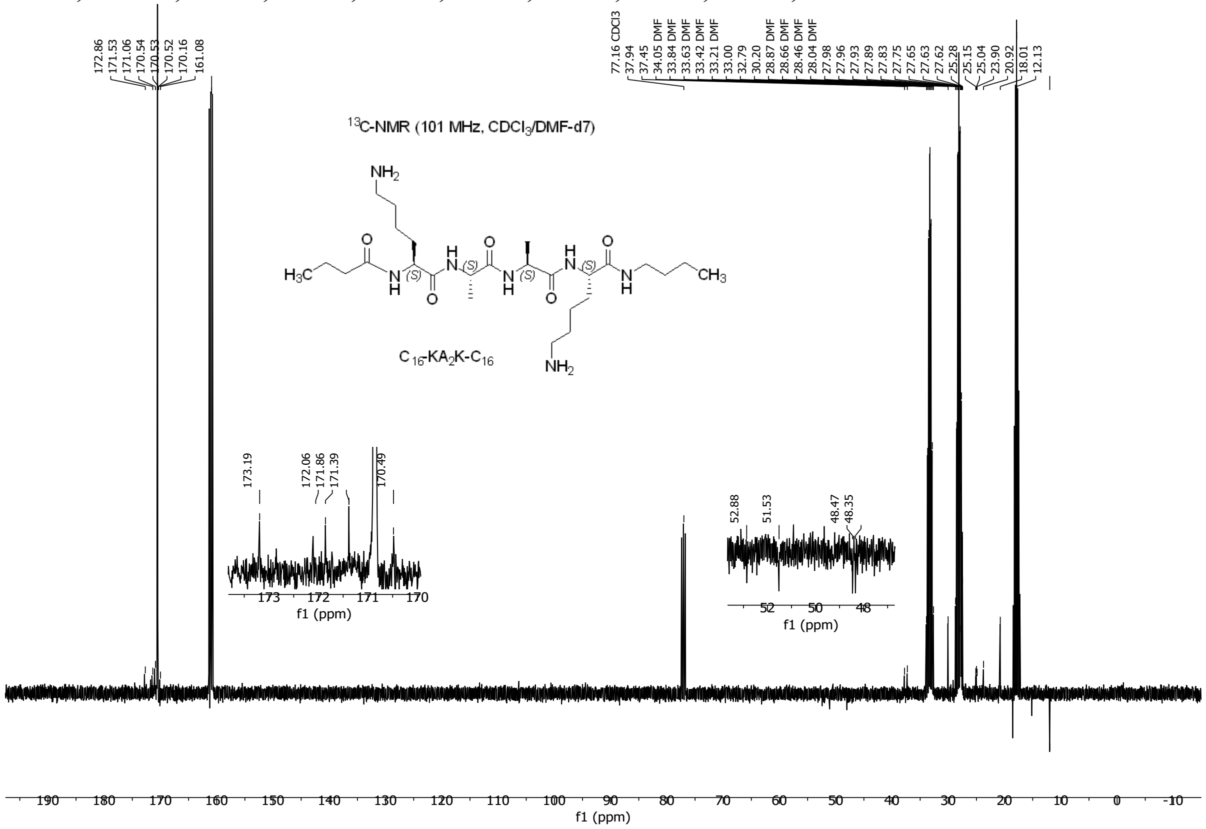

Figure S11. ^{1}H NMR of 3a (C_{16}-KGK-C_{16})

^{1}H NMR (400 MHz, Acetic Acid-d4) δ 4.59 – 4.53 (m, 2H), 4.04 (d, *J* = 4.3 Hz, 2H), 3.24 (t, *J* = 7.2 Hz, 2H), 3.09 – 3.02 (m, 4H), 2.34 – 2.27 (m, 2H), 1.91 – 1.81 (m, 2H), 1.78 – 1.65 (m, 4H), 1.62 – 1.56 (m, 2H), 1.53 – 1.44 (m, 4H), 1.27 (s, 54H), 0.94 – 0.83 (m, 6H).

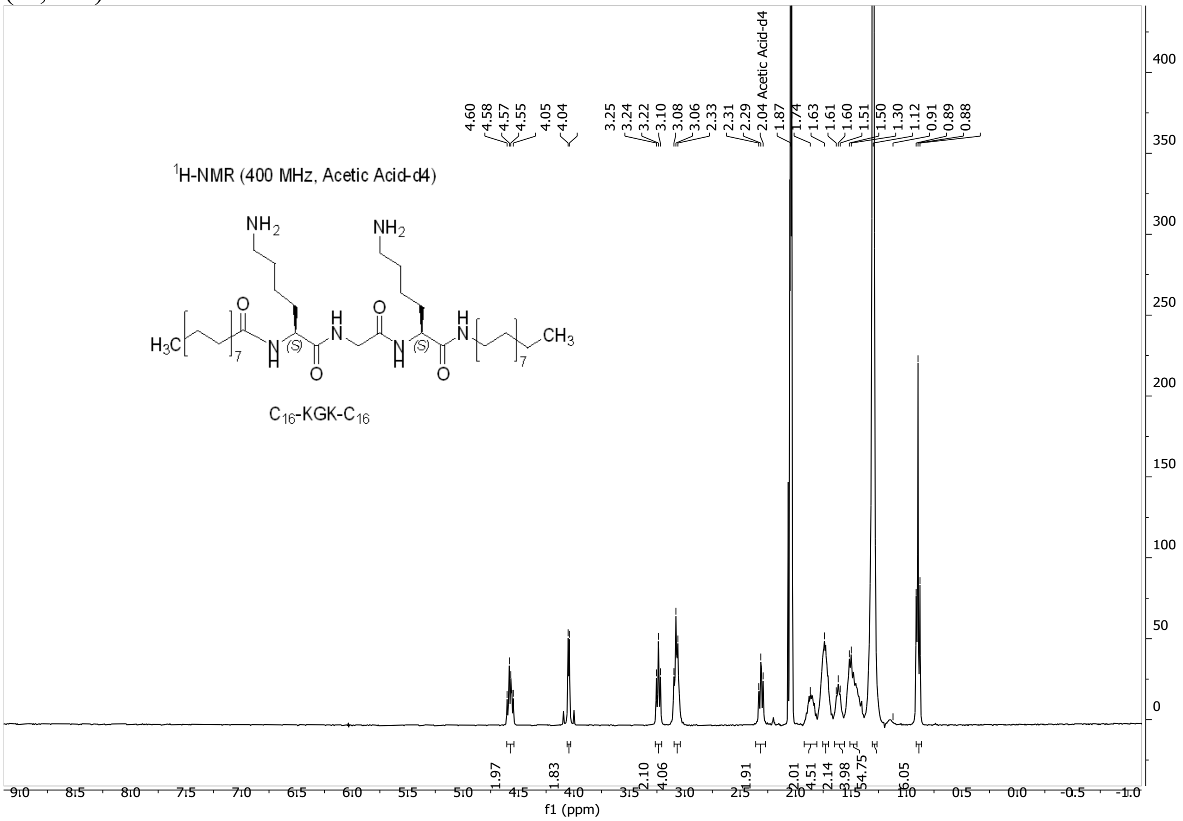

Figure S12. ^{13}C NMR of 3a (C$_{16}$-KGK-C$_{16}$)

^{13}C NMR (APT mode, 101 MHz, MeOD /Acetic acid-d4) δ 177.12, 175.38, 173.83, 171.81, 52.11, 51.89, 51.66, 51.44, 51.22, 43.71, 40.70, 36.67, 32.90, 32.23, 31.84, 30.66, 30.64, 30.61, 30.58, 30.51, 30.32, 30.28, 30.23, 30.09, 27.80, 27.76, 27.55, 26.66, 23.59, 23.51, 23.44, 14.46.

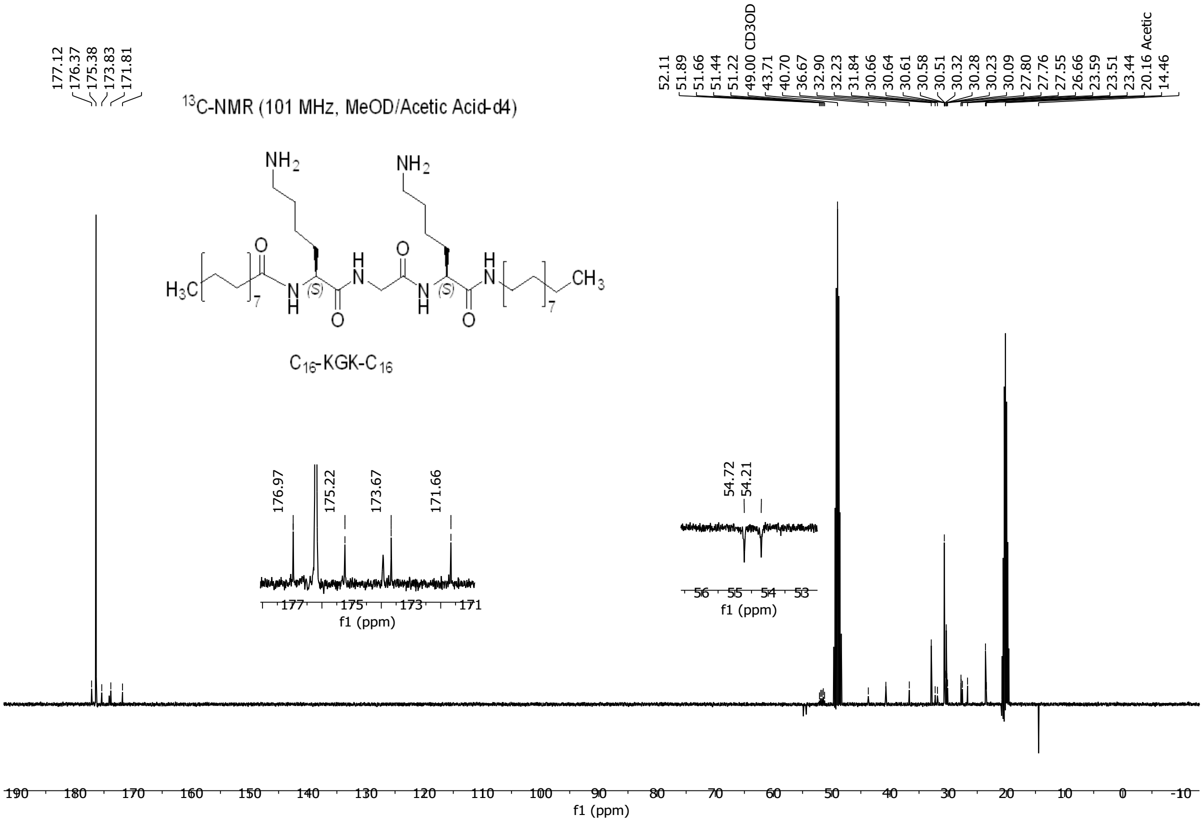

Figure S13. ^{1}H NMR of 3b (C_{16}-KG$_2$K-C_{16})

^{1}H NMR (500 MHz, Acetic acid-d4) δ 4.56 (td, J = 9.0, 5.5 Hz, 2H), 4.08 (s, 2H), 4.04 (s, 2H), 3.23 (h, J = 6.7 Hz, 2H), 3.07 (td, J = 7.4, 2.4 Hz, 4H), 2.32 (dt, J = 8.9, 4.1 Hz, 2H), 1.87 – 1.80 (m, 1H), 1.79 – 1.66 (m, 4H), 1.64 – 1.56 (m,1H), 1.55 – 1.41 (m, 4H), 1.30 (s, 48H), 0.93 – 0.85 (m, 6H).

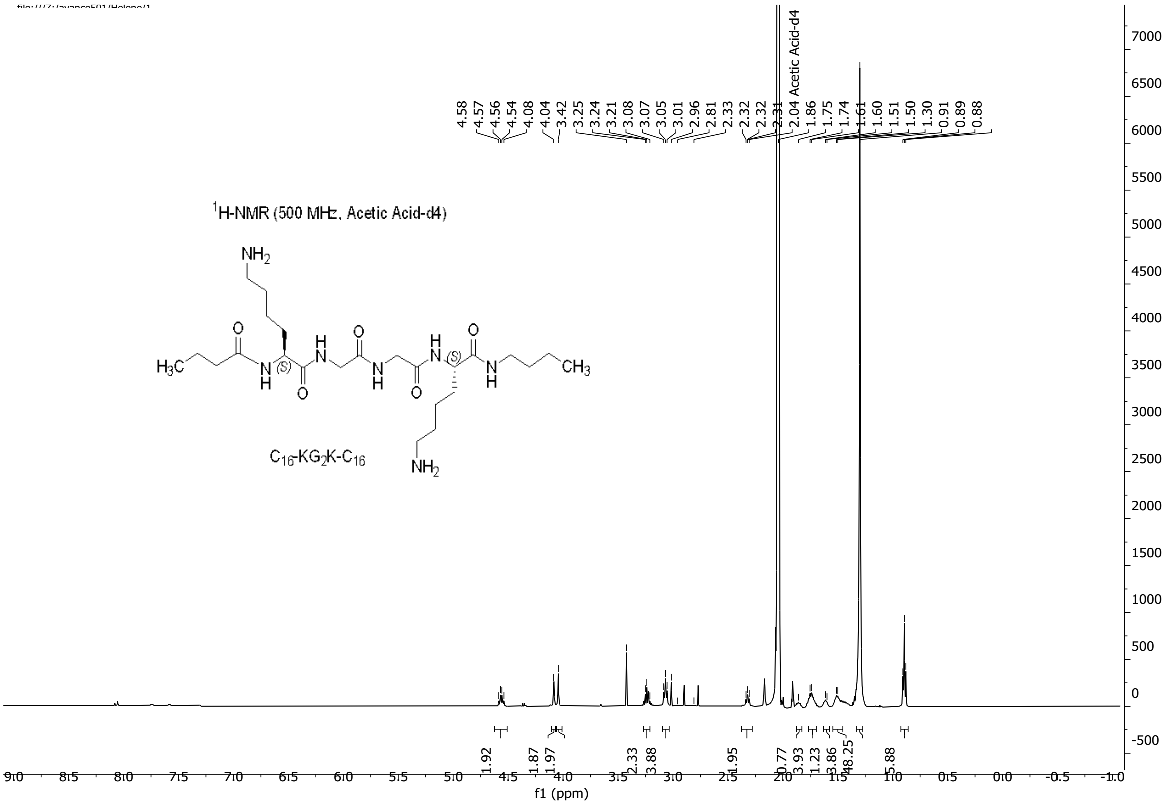

Figure S14. ^{13}C NMR of 3b (C_{16}-KG_2K-C_{16})

^{13}C NMR (APT mode, 101 MHz, Acetic acid-d4) δ 176.39, 174.85, 174.76, 173.82, 173.09, 173.01, 44.68, 41.83, 41.71, 37.65, 33.81, 32.94, 31.60, 31.59, 31.57, 31.55, 31.54, 31.50, 31.45, 31.25, 31.19, 31.14, 30.90, 28.72, 28.48, 28.28, 27.61, 24.50, 24.26, 24.20, 15.44.

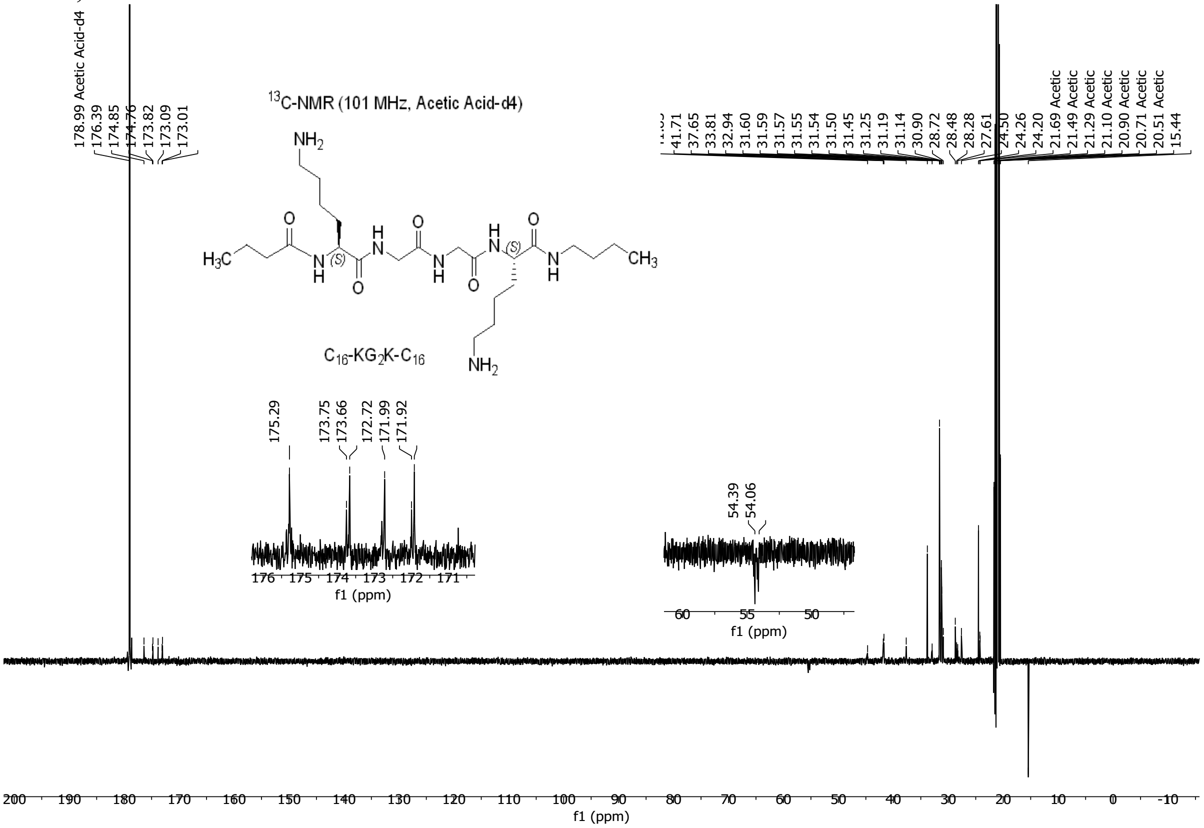

References (Supporting Material)

S1) I. E. Gouvea, M. A. Izidoro, W. A. S. Judice, M. H. S. Cezari, G. Caliendo, V. Santagada, C. N. D. dos Santos, M. H. Queiroz, M. A. Juliano, P. R. Young, D. P. Fairlie, L. Juliano, "Substrate specificity of recombinant dengue 2 virus NS2B-NS3 protease: Influence of natural and unnatural basic amino acids on hydrolysis of synthetic fluorescent substrates." *Arch. Biochem. Biophys.* 457, 187–196 (2007).

S2) D. Leung, K. Schroder, H. White, N.-X. Fang, M. J. Stoermer, G. Abbenante, L. J. Martin, P. R. Young, D. P. Fairlie, "Activity of Recombinant Dengue 2 Virus NS3 Protease in the Presence of a Truncated NS2B Co-factor, Small Peptide Substrates, and Inhibitors." *J. Biol. Chem.* 276, 45762-45771 (2001).

S3) M. M. Kacprzak, J. R. Peinado, M. E. Than, J. Appel, S. Henrich, G. Lipkind, R. A. Houghten, W. Bode, I. Lindberg, "Inhibition of furin by polyarginine-containing peptides: nanomolar inhibition by nona-D-arginine." *J. Biol. Chem.* 279, 36788–36794 (2004).

S4) T. A. Ayoubi, J. W. Creemers, A. J. Roebroek, W. J. van de Ven, "Expression of the dibasic proprotein processing enzyme furin is directed by multiple promoters." *J. Biol. Chem.* 269, 9298-9303 (1994).
S5) I. Lindberg, Y. Zhou, "Overexpression of neuropeptide precursors and processing enzymes." *Methods in Neuroscience,* 23, 94–108 (1995), Academic Press, Orlando, FL
S6) A. Cameron, J. Appel, R. A. Houghten, I. Lindberg, "Polyarginines are potent furin inhibitors." *J. Biol. Chem.* 275, 36741–36749 (2000).
S7) F. Jean, K. Stella, L. Thomas, G. Liu, Y. Xiang, A. J. Reason, G. Thomas, "α_1-Antitrypsin Portland, a bioengineered serpin highly selective for furin: application as an antipathogenic agent." *Proc. Natl. Acad. Sci. USA* 95, 7293–7298 (1998).
S8) M. Damen, J. Aarbiou, S. F. M. van Dongen, R. M. Buijs-Offerman, P. P. Spijkers, M. van den Heuvel, K. Kvashnina, R. J. M. Nolte, B. J. Scholte, M. C. Feiters, "Delivery of DNA and siRNA by novel gemini-like amphiphilic peptides." *J. Controlled Release* 145, 33–39 (2010).
